# Childhood adversity and mental health admission patterns prior to young person suicide (CHASE): a case-control 36 year linked hospital data study, Scotland UK 1981–2017

**DOI:** 10.1192/bjo.2024.69

**Published:** 2024-06-03

**Authors:** Nadine Dougall, Jan Savinc, Margaret Maxwell, Thanos Karatzias, Rory C. O'Connor, Brian Williams, Ann John, Helen Cheyne, Claire Fyvie, Jonathan I. Bisson, Carina Hibberd, Susan Abbott-Smith, Liz Nolan, Jennifer Murray

**Affiliations:** School of Health & Social Care, Edinburgh Napier University, UK; Nursing, Midwifery and Allied Health Professions Research Unit, University of Stirling, UK; Institute of Health & Wellbeing, University of Glasgow, UK; School of Health, Social Care & Life Sciences, University of the Highlands & Islands, UK; Department of Population Psychiatry, Suicide and Informatics, Swansea University Medical School, UK; and Public Health Wales, Cardiff, UK; The Rivers Centre, NHS Lothian, Edinburgh, UK; Cardiff University School of Medicine, Cardiff University, UK; Faculty of Health Sciences & Sport, University of Stirling, UK; Child and Adolescent Mental Health Service (CAMHS), NHS Lothian, Edinburgh, UK; Aberlour, Scotland's children's charity (SC007991), Stirling, UK

**Keywords:** Childhood adversity, adverse childhood experiences, mental health, self-harm, suicide

## Abstract

**Background:**

Childhood adversity is associated with increased later mental health problems and suicidal behaviour. Opportunities for earlier healthcare identification and intervention are needed.

**Aim:**

To determine associations between hospital admissions for childhood adversity and mental health in children who later die by suicide.

**Method:**

Population-based longitudinal case-control study. Scottish in-patient general and psychiatric records were summarised for individuals born 1981 or later who died by suicide between 1991 and 2017 (cases), and matched controls (1:10), for childhood adversity and mental health (broadly defined as psychiatric diagnoses and general hospital admissions for self-harm and substance use).

**Results:**

Records were extracted for 2477 ‘cases’ and 24 777 ‘controls’; 2106 cases (85%) and 13 589 controls (55%) had lifespan hospitalisations. Mean age at death was 23.7; 75.9% were male. Maltreatment or violence-related childhood adversity codes were recorded for 7.6% cases aged 10–17 (160/2106) versus 2.7% controls (371/13 589), odds ratio = 2.9 (95% CI, 2.4–3.6); mental health-related admissions were recorded for 21.7% cases (458/2106), versus 4.1% controls (560/13 589), odds ratio = 6.5 (95% CI, 5.7–7.4); 80% of mental health admissions were in general hospitals. Using conditional logistic models, we found a dose-response effect of mental health admissions <18y, with highest adjusted odds ratio (aOR) for three or more mental health admissions: aOR_male_ = 8.17 (95% CI, 5.02–13.29), aOR_female_ = 15.08 (95% CI, 8.07–28.17). We estimated that each type of childhood adversity multiplied odds of suicide by aOR_male_ = 1.90 (95% CI, 1.64–2.21), aOR_female_ = 2.65 (95% CI, 1.94–3.62), and each mental health admission by aOR_male_ = 2.06 (95% CI, 1.81–2.34), aOR_female_ = 1.78 (95% CI, 1.50–2.10).

**Conclusions:**

Our lifespan study found that experiencing childhood adversity (primarily maltreatment or violence-related admissions) or mental health admissions increased odds of young person suicide, with highest odds for those experiencing both. Healthcare practitioners should identify and flag potential ‘at-risk’ adolescents to prevent future suicidal acts, especially those in general hospitals.

Suicide is a major cause of death for young people worldwide with wide-ranging impacts relevant to public health. Broadly speaking, deaths by suicide in Scotland have been increasing since 2014 after a period of decline since the 1990s,^1,2^ with notable increases in the 15–24 age bracket, and a decrease during the COVID-19 pandemic in 2020. Deaths by suicide are relatively rare outcomes, particularly in children, but they are indicative of a larger population vulnerable to suicide with self-harm and mental ill health being known risk factors for suicide.^3,4^ This population vulnerability is a growing problem with the prevalence of both self-harm and poor mental health increasing in recent years.^3,5,6^

Childhood adversity has also been reported as a risk factor for suicide,^7–9^ with a confirmed strong dose-response relationship between types and ‘dose’ of adversity.^10,11^ However, these associations have yet to be confirmed with a lifespan health data study that can explore associations between childhood adversity and future suicidal behaviour. Previous studies have found higher rates of service use, including hospitalisations, in children who had experienced adversity.^12,13^ Understanding health service contacts for childhood adversity unaffected by recall bias may provide opportunities for earlier suicide prevention interventions in childhood and adolescence before suicidal behaviour emerges. We hypothesised that people who died by suicide had more frequent admissions for childhood adversity and mental health, including admissions for self-harm. Therefore, the aim of this study was to investigate patterns of lifespan hospital contacts for childhood adversities, mental health status and suicidal behaviour prior to later young person suicide.

## Ethics and consent statement

The study was reviewed and approved by the North of Scotland Research Ethics Committee 1 (REC) on 4 May 2018 with REC reference 18/NS/0054. Consent to use the de-identified data was provided by the Public Benefit and Privacy Panel (PBPP) for Health and Social Care on 6 September 2018 with reference 1617-0228. In accordance with the obtained approvals, all data were de-identified, and individuals’ informed consent was not necessary.

## Method

### Research questions

This analysis aimed to report to three pre-specified research questions (RQ) in the study protocol:^14^ (1) What is the relationship between number and type of childhood adversities and suicide, stratified by age and sex (assigned at birth)? (2) What is the relationship between type and number of childhood adversities, subsequent mental health and self-harm admissions prior to suicide? (3) Can a dose-response relationship of number of childhood adversities with suicidal behaviour be confirmed, and can type of childhood adversity be ranked as having impact on later life?

### Study design and participants

A retrospective matched case-control design was employed as an efficient way of studying ‘rare’ events while holding constant the effects of age, sex and geographical location, and minimising resource and data use,^15^ a design adopted elsewhere.^16,17^ This study made use of NHS data captured electronically from 1981 in Scotland, a uniquely valuable administrative data-set in the world containing lifespan data from birth to young adult suicide.^18^ ‘Cases’: linked lifespan hospitalisation records were extracted for individuals born in Scotland from 1981 onwards who later died by suicide or events of undetermined intent^19^ aged 10 years or older (see Supplementary file available at https://doi.org/10.1192/bjo.2024.69) during the period 1991–2017. ‘Controls’ were drawn from the same time period; they were randomly matched to ‘cases’, in a 1:10 ratio, based on postcode (residence at time of death by suicide), sex assigned at birth, and birth year, from a general population sample (see Supplementary Tables 3 and 4 for details). Postcode was included in matching to control for area-level deprivation (see Supplementary Tables 5 and 6), previously associated with poor mental health and suicide.^6,17,20,21^ Data were linked using the NHS Community Health Index (CHI) to collate individuals’ births, death records and lifetime in-patient and day-case hospital admissions for physical and mental health. Maternal death records were also linked using the mothers’ CHI identifiers in birth records. Details of the design and linkage are available in the published protocol.^14^ Results were reported according to the ‘REporting of studies Conducted using Observational Routinely-collected health Data’ (RECORD) statement.^22^ Study investigators included academics, clinicians and charitable sector experts representing people with lived experience.

### Childhood adversity and mental health codes

As defined in the protocol,^14^ adverse events were identified in hospital admissions occurring before age 18 years, based on International Classification of Disease (ICD) coded diagnoses (ICD-10 was in use from 1 April 1997, and prior to that ICD-9; see Supplementary Table 1). All recorded diagnoses were used to code admissions, including the ‘main’ and up to five ‘other’ diagnoses, as well as diagnoses at admission, or discharge, in psychiatric records. Adverse events were operationalised in two ways: (1) maltreatment or violence-related codes (MVR)^23,24^ for admissions before age 18, or (2) codes *suggestive* of maltreatment^25^ for age <10. Mental health-related (mental health) codes were defined as codes listed in Clinical Classifications Software (CCS):^26–28^this covered ICD-9 and ICD-10 Chapter V (Mental and behavioural disorders), ICD-9 codes E950-E959 and ICD-10 codes X60-X84, Y870 for Self-harm (see Supplementary Table 2), as well as individual codes in other chapters; mental ill health was thus broadly defined, including self-harm and alcohol- and substance-use diagnoses (see Supplementary Table 9 for the most frequent diagnoses, and Supplementary spreadsheet for complete list of inclusions). We used CCS as a semi-automated way to harmonise ICD-9 and ICD-10 codes through time. An additional rationale for using the CCS broad mental health definition was that by capturing a wide range of admissions, we might identify the best opportunities by admission type or pattern for healthcare staff to intervene earlier with suicide prevention measures.

In addition to the protocol-defined codes we included: codes indicating poisoning (accidental and ‘undetermined intent’); hospitalisations with residence codes in the ‘Admission from’ or ‘Discharge to’ fields, indicative of homelessness or ‘care experience’ (codes indicating foster care, care homes or residential institutions; see Supplementary Table 7). Maternal death records were used to determine maternal bereavement before age 18. Details of coding are in the Supplementary file, Supplementary spreadsheet and on GitHub at https://github.com/jsavinc/CHASe-outside-safe-haven.

### Statistical analysis

All analyses were performed using R (version 3.6.1) for Windows using ‘tidyverse’, ‘lubridate’ and ‘fuzzyjoin’ packages.^29–31^ Descriptive statistics summarised group characteristics. Area-level deprivation measures (Carstairs Index,^32^ Scottish Index of Multiple Deprivation (SIMD),^33,34^ Urban–rural indicator^35^) were derived from death record postcodes for cases, and CHI records for controls at the date of linked cases’ deaths. Controls’ hospitalisation data were censored at an age (in days) equivalent to their matched cases’ age at death.

Most childhood adversity categories were relatively rare, so frequencies were summarised by age at first event (admission to hospital) into two periods, informed by the studies of MVR^23,24^ and codes suggestive of maltreatment:^25^ childhood (age <10 years) and adolescence (≥10 and <18 years). Relative order of adverse events and mental health diagnoses was determined for individuals <18 years using frequencies and odds ratios. Two conditional logistic regression models were fitted using suicide as outcome, stratified by sex assigned at birth. Model 1 included categorical variables for type and number of adverse events and mental health diagnoses prior to age 18, as explanatory variables to ascertain the relative strength of associations with suicide of different adverse events and mental health admissions. Model 2 tested for an interaction between adverse events and mental health admissions, and included continuous variables for number of types of adverse events, number of mental health admissions before age 18 and their interaction. Age and postcode-derived variables were excluded from the models because of being constant within each stratum of cases and controls as a result of case-control matching, and consequently non-estimable.

## Results

### Demographic data

**Fig. 1:**
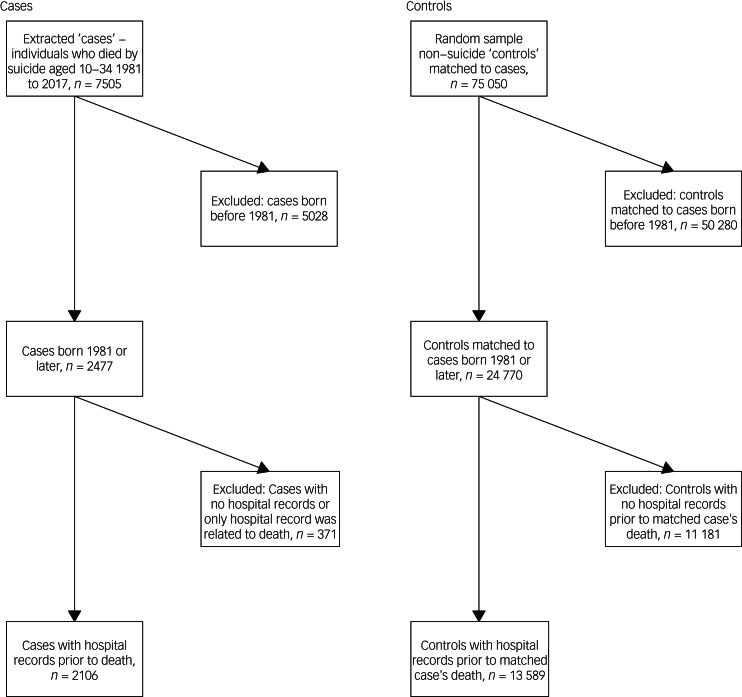
Data inclusion and exclusion flowchart.

Data were extracted on 7505 individuals who died by suicide in Scotland (‘cases’) in the period 1991–2017, of whom 2477 were born in 1981 or later and potentially had lifetime records available (see Fig. 1 for summary of exclusions; Table 1 for characteristics of cohort). As we were interested in hospital contacts to understand healthcare suicide prevention intervention opportunities, individuals with no lifetime in-patient admissions or a single death-coded record were excluded (*N*_cases_ = 371; *N*_controls_ = 11 181; see Discussion for effects on odds ratios. Conversely, 85.0% (2106/2477) of cases had accrued lifespan in-patient records and were defined as the study population, with a corresponding 54.9% (13 589/24 770) controls (see Table 1 for demographics). Males numbered 75.9% (1599/2106) cases and 77.5% (10 537/13 589) controls. Mean age at death was 23.7 years (s.d. = 4.9) for cases with lifespan records and 21.9 years (s.d. = 4.9) for cases without, i.e. those in contact with hospital services remained alive for 1.8 (95% CI, 1.25–2.34) years longer on average (*t* = 6.46, d.f. = 2,475, *P* < 0.001). There was a deprivation gradient, with 37.8 and 29.4% of cases in the most deprived quintile for SIMD and Carstairs Index variables, respectively. Most cases (88.6%) and controls (87.9%) lived in urban areas of Scotland using NRS 2-fold classification,^35^ slightly more than 83% of the corresponding national population data. Psychiatric in-patient admissions represented 11.6% of lifetime in-patient admissions accrued by cases, a factor of 8.3 times more than the 1.4% accrued by controls (see Table 2). Cases accrued more diagnoses from ICD chapters for ‘Mental disorders’ and ‘Injury and poisoning’ (with associated ‘External causes’) in their lifetime records than controls (see Supplementary Table 8).

**Table 1:** Characteristics of the overall study population: ‘cases’ (death by suicide)

		All	Male	Female
Age	Mean (s.d.)	22.9 (4.99)	23.1 (4.90)	22.4 (5.23)
	*n* (%)	2477 (100%)	1883 (76.0%)	594 (24.0%)
Age group	*n* (%)			
≥10 to 14		75 (3.0%)	48 (2.5%)	27 (4.5%)
≥15 to 19		627 (25.3%)	450 (23.9%)	177 (29.8%)
≥20 to 24		840 (33.9%)	659 (35.0%)	181 (30.5%)
≥25 to 30		657 (26.5%)	509 (27.0%)	148 (24.9%)
≥30 to 34		278 (11.2%)	217 (11.5%)	61 (10.3%)
Not in work^a^		1054 (42.6%)	738 (39.2%)	316 (53.2%)
Carstairs Index (quintile)	Mean (s.d.)	2.54 (1.34)	2.56 (1.45)	2.48 (1.33)
1 (most deprived)	*n* (%)	723 (29.4%)	533 (28.5%)	190 (32.3%)
2		604 (24.6%)	473 (25.3%)	131 (22.2%)
3		487 (19.8%)	370 (19.8%)	117 (19.9%)
4–5		645 (26.2%)	494 (26.4%)	151 (25.6%)
SIMD (quintile)	Mean (s.d.)	2.34 (1.38)	2.37 (1.35)	2.25 (1.32)
1 (most deprived)	*n* (%)	929 (37.8%)	688 (36.8%)	241 (40.9%)
2		545 (22.2%)	418 (22.4%)	127 (21.6%)
3		438 (17.8%)	332 (17.8%)	106 (18.0%)
4		308 (12.5%)	244 (13.1%)	64 (10.9%)
5		239 (9.7%)	188 (10.1%)	51 (8.7%)
Urban–rural index	*n* (%)			
Large urban areas		871 (35.4%)	652 (34.9%)	219 (37.2%)
Other urban areas		1008 (41.0%)	764 (40.9%)	244 (41.4%)
Accessible small towns		209 (8.5%)	154 (8.2%)	55 (9.3%)
Remote, very remote small towns, and accessible, remote, very remote rural		371 (15.1%)	300 (16.0%)	71 (12.1%)

SIMD, Scottish Index of Multiple Deprivation.

a. National Records Scotland death registrations category of ‘students, independent means, no occupation or disability’. Demographic data were derived from death registrations. The Carstairs Index is scored from 1 to 5 from most deprived to least deprived; scores of 4–5 were merged because of small numbers. The 2016 8-fold urban–rural index was used, with remote and very remote small towns, and all rural areas merged because of small numbers. Controls were matched to ‘cases’ and comprised *N* = 24 770 individuals overall, of whom *n* = 18 830 and *n* = 5940 were male and female, respectively.

**Table 2:** Characteristics of ‘cases’ and ‘controls’, with or without lifetime hospital admissions

Cohort		Cases						Controls					
		With lifetime hospital admissions			No lifetime hospitalisations			With lifetime hospital admissions			No lifetime hospitalisations		
Sex		Both	Male	Female	Both	Male	Female	Both	Male	Female	Both	Male	Female
*n*		2106	1599	507	371	284	87	13 589	10 537	3052	11 181	8293	2888
Proportion of extracted records (cases/controls)^1^	%	85.0	64.6	20.5	15.0	11.5	3.5	54.9	42.5	12.3	45.1	33.5	11.7
Proportion of cohort (cases/controls) with or without records	%	100	75.9	24.1	100	76.5	23.5	100	77.5	22.5	100	74.2	25.8
Age at death	Mean (s.d.)	23.65 (4.93)	23.79^1^ (4.86)	23.19^1^ (5.15)	21.85 (4.94)	22.19^1^ (4.82)	20.77^1^ (5.18)	NA	NA	NA	NA	NA	NA
Carstairs indicator quintile (1 = most deprived)	Mean (s.d.)	2.49 (1.33)	2.51 (1.33)	2.44^2^ (1.33)	2.80^††^ (1.38)	2.84^††^ (1.40)	2.69 (1.30)	2.52 (1.31)	2.54** (1.32)	2.43** (1.30)	2.56^††^ (1.37)	2.57^††^ (1.38)	2.53 (1.35)
	Median (IQR)	2 (1; 4)	2 (1; 4)	2 (1; 3)	3 (2; 4)	3 (2; 4)	3 (2; 4)	2 (1; 3)	2 (1; 4)	2 (1; 3)	2 (1; 4)	2 (1; 4)	2 (1; 4)
SIMD quintile (1 = most deprived)	Mean (s.d.)	2.27 (1.31)	2.30^1^ (1.33)	2.16^1^ (1.27)	2.77^††^ (1.46)	2.76^††^ (1.45)	2.81^††^ (1.49)	2.29 (1.32)	2.33** (1.33)	2.16** (1.28)	2.40^††^ (1.38)	2.43**^††^ (1.38)	2.34**^††^ (1.35)
	Median (IQR)	2 (1; 3)	2 (1; 3)	2 (1; 3)	3 (1; 4)	3 (1; 4)	3 (1; 4)	2 (1; 3)	2 (1; 3)	2 (1; 3)	2 (1; 3)	2 (1; 3)	2 (1; 3)
Urban–rural indicator: Large urban areas^3^	*n* (%)	735 (34.9%)			136 (36.7%)			4018 (29.6%)			4692 (42.0%)		
Other urban areas		869 (41.3%)			139 (37.5%)			6057 (44.6%)			4023 (36.0%)		
Accessible small towns		180 (8.5%)			29 (7.8%)			1265 (9.3%)			825 (7.4%)		
Remote small towns		46 (2.2%)			≤10			312 (2.3%)			208 (1.9%)		
Very remote small towns		26 (1.2%)			≤10			187 (1.4%)			133 (1.2%)		
Accessible rural		162 (7.7%)			28 (7.5%)			1131 (8.3%)			769 (6.9%)		
Remote rural		36 (1.7%)			≤10			236 (1.7%)			184 (1.6%)		
Very remote rural		43 (2.0%)			12 (3.2%)			280 (2.1%)			270 (2.4%)		
Not in work^4^	%	42.4	38.9**	53.6**	43.9	41.2**	52.9**	NA	NA	NA	NA	NA	NA
Hospitalisations	*n*	13 534	9,269	4265	NA	NA	NA	46 403	35 715	10 688	NA	NA	NA
General hospital (SMR01)	*n*	11 965	8,270	3695	NA	NA	NA	45 750	35 249	10 501	NA	NA	NA
	%	88.4	89.2	86.6	NA	NA	NA	98.6	98.7	98.3	NA	NA	NA
Psychiatric (SMR04)	*n*	1569	999	570	NA	NA	NA	653	466	187	NA	NA	NA
	%	11.6	10.8	13.3	NA	NA	NA	1.4	1.3	1.7	NA	NA	NA

a. Proportion of *N* = 2477 for cases and *N* = 24 770 for controls. b. NRS death registrations category of ‘students, independent means, no occupation, disability’ – not available for controls; medians (IQR) reported where data are skewed.

IQR, interquartile range; NA, not applicable; SIMD, Scottish Index of Multiple Deprivation.

1 *P < 0.05 or **P < 0.01 significantly different between sexes at 95% or 99% threshold, respectively.

2 ^†^*P* < 0.05 or ^††^*P* < 0.01 significantly different between cases & controls at 95% or 99% threshold, respectively.

3 Urban–rural indicator not reported for males and females separately because of small numbers.

4 NRS death registrations category of ‘students, independent means, no occupation, disability’ – not available for controls; medians (IQR) reported where data are skewed.

### Number and type of admissions for adversity and mental health

All the following reported odds ratios were statistically significant at *P* < 0.05 and include a 95% CI, in line with the recognition of the limitations and misinterpretations associated with *P*-values. In all age groups, cases had disproportionally more in-patient admissions coded for adversities and mental health admissions than controls (see Table 3).

**Table 3:** Childhood adversity by age at first diagnosis (index admissions), sex and case status

			Cases			Controls			Odds ratio (95% CI)		
Adversity	Sex		Both	Male	Female	Both	Male	Female	Both	Male	Female
	Age	*n*	2106	1599	507	13 589	10 537	3052	−	−	−
MVR	<10	*n* (%)	35 (1.7%)	23 (1.4%)	12 (2.4%)	140 (1.0%)	106 (1.0%)	34 (1.1%)	1.62 (1.12–2.36)	1.44 (0.91–2.26)	2.15 (1.11–4.18)
	≥10, <18	*n* (%)	160 (7.6%)	121 (7.6%)	39 (7.7%)	371 (2.7%)	325 (3.1%)	46 (1.5%)	2.93 (2.42–3.55)	2.57 (2.07–3.19)	5.45 (3.51–8.44)
Codes suggestive of maltreatment or neglect (Schnitzer et al)^25^	<10	*n* (%)	307 (14.6%)	228 (14.3%)	79 (15.6%)	2042 (15.0%)	1576 (15.9%)	466 (15.3%)	0.96 (0.85–1.10)	0.95 (0.81–1.10)	1.02 (0.79–1.33)
Codes suggestive of maltreatment or neglect (Schnitzer et al),^25^ excluding dental caries	<10	*n* (%)	74 (3.5%)	53 (3.3%)	21 (4.1%)	366 (2.7%)	301 (2.9%)	65 (2.1%)	1.32 (.02–1.70)	1.17 (0.87–1.57)	1.99 (1.20–3.28)
Mental health-related diagnosis	≥10, <18	*n* (%)	458 (21.7%)	282 (17.6%)	176 (34.7%)	560 (4.1%)	384 (3.6%)	176 (5.8%)	6.47 (5.66–7.39)	5.66 (4.80–6.67)	8.69 (6.85–11.03)
Maternal death	<18	*n* (%)	58 (2.8%)	18 (3.6%)	40 (2.5%)	188 (1.4%)	29 (1.0%)	159 (1.5%)	2.02 (1.50–2.72)	1.67 (1.18–2.38)	3.84 (2.11–6.96)
Place of residence indicating care experience	<18	*n* (%)	62 (2.9%)			53 (0.4%)			7.75 (5.35–11.21)		

MVR, maltreatment and violence-related; frequencies of individuals with MVR diagnosis, diagnosis suggestive of maltreatment (Schnitzer), and mental health-related diagnosis, or experience of maternal death. Age group was determined at the individual's first diagnosis of each kind of adversity. Diagnoses in any position were included. Admissions related to death were excluded. The denominator is the number of ‘cases’ or ‘controls’ with records prior to death, broken down by sex, respectively. Note that codes suggestive of maltreatment or neglect were only defined for ages <10. Mental health-related diagnoses before age 10 were very rare and are not shown. There is a small degree of overlap between MVR, codes suggestive of maltreatment or neglect, and mental health-related diagnoses, specifically with respect to rarely used external cause codes for events of undetermined intent. Maternal death was counted only for individuals with hospital records prior to death, and only when the mother died prior to the death of the individual (or the equivalent date in controls); not all individuals could be linked to their mothers’ records so this is likely an underestimate of bereavement; however, the same denominator was used as for other types of adversity for comparison. Place of residence indicating care experience was not reported separately by sex due to small numbers.

Maltreatment and violence-related (MVR) admissions were rarely recorded before age 10, with 1.7% cases (35/2106) versus 1.0% controls (140/13 589), odds ratio = 1.62 (95% CI, 1.12–2.36); in 10–17 year-olds they were found for 7.6% cases (160/2106) versus 2.7% controls (371/13 589), odds ratio = 2.93 (95% CI, 2.42–3.55). Using the larger denominator of individuals with or without lifetime hospitalisations had the effect of increasing the odds ratios (see Discussion).

Mental health admissions for those under 10 were too sparse to report. Mental health admissions aged 10–17 were more common, found in 21.7% cases (458/2106) versus 4.1% of controls (560/13 589), a factor of 5.3 times higher and odds ratio = 6.47 (95% CI, 5.66–7.39). Cases had a higher rate of mental health diagnoses than controls (see Supplementary Table 9 for breakdown by diagnosis types) – this was particularly acute for female cases where 29.6% (150/507) had an intentional self-inflicted injury diagnosis in in-patient records (combined general and psychiatric hospital) before age 18, odds ratio = 11.91 (95% CI, 9.06–15.66). Self-inflicted injury and alcohol-related disorders were the most common mental health-related diagnoses. Approximately 80% of mental health admissions were recorded in general hospitals.

Maternal death before age 18 was more common for cases with 2.7% (58/2106) affected, compared with 1.4% controls (188/13 589), odds ratio = 2.02 (95% CI, 1.50–2.72). Admissions coded with a place of residence indicative of ‘care experience’ before age 18 more were found for 2.9% cases (62/2106), versus 0.4% controls (53/13 589), odds ratio = 7.75 (95% CI, 5.5–11.21). Very few individuals with place of residence were coded as ‘no fixed abode’, so this code was combined with care-related codes before age 18 to form a single place of residence-related adversity variable in Models 1 and 2.

MVR and mental health-related codes included poisoning diagnoses, so we investigated poisonings by intent (see Supplementary Table 10). Intentional poisonings (included under the mental health category of ‘Self-inflicted injury’) were more common in cases than controls, with 27.4% female cases (139/507) and 8.0% male cases (128/1599) having such diagnoses, compared with 3.2% female controls (98/3052) and 0.8% male controls (87/10 537); odds ratio_female_ = 11.39 (95% CI, 8.60–15.07) and odds ratio_male_ = 10.45 (95% CI, 7.92–13.80). For data relating to admissions aged <10, accidental and undetermined intent poisonings were combined because of small numbers and were more common with 4.0% cases (84/2106) compared with 2.8% controls, (374/13 589), odds ratio = 1.47 (95% CI, 1.15–1.87). This ratio increased in individuals aged 10 to 17, with 3.3% of cases (69/2106) having such diagnoses compared with 0.4% of controls (57/13 589), odds ratio = 8.04 (95% CI, 5.64–11.46).

Codes suggestive of maltreatment or neglect before age 10 occurred more frequently but were similar between groups with 14.6% cases (307/2106) versus 15.0% controls (2042/13 589), odds ratio = 0.96 (95% CI, 0.85–1.10). These proportions decreased to 3.5% cases (74/2106) and 2.7% controls (366/13 589), odds ratio = 1.32 (95% CI, 1.02–1.70) when we excluded dental caries diagnoses (see Supplementary file).

### Relative order of hospital presentation for adverse events and mental health diagnoses

Frequencies and odds ratios for the relative impact of order of first presentation for adverse events and/or mental health along the lifespan were estimated (see Table 4). Of the possible admission combinations, in order of decreasing frequency, the proportion of cases (combined sexes) had lifetime admissions as follows: for ‘mental health only’ 13.5% (286/2106); for ‘adverse event admissions only’ 9.5% (201/2106); for ‘adverse events admissions first, mental health admission second’ 3.8% (81/2106); both co-occurring (within 28 days) 2.9% (62/2106); ‘mental health admission first, adverse event second’ 2.4% (50/2106). In individuals who experienced both adverse events and mental health admissions, adversity before mental health admission was generally more frequent than mental health admission first followed by adverse event, and the difference in frequency was more pronounced in females, though the number of individuals affected was very small.

**Table 4:** Frequencies and odds ratios of relative order of adverse events and mental health-related diagnoses in individuals aged <18

Sex	Order of events	Cases, *N* (%)	Controls, *N* (%)	Odds ratio (95% CI)
Both	Adversity, then MH	81 (3.8%)	64 (0.5%)	8.45 (6.07–11.77)
	MH, then Adversity^a^	50 (2.4%)	40 (0.3%)	8.24 (5.42–12.52)
	Both simultaneously	62 (2.9%)	72 (0.5%)	5.69 (4.04–8.02)
	Adversity only	201 (9.5%)	972 (7.2%)	1.37 (1.17–1.61)
	MH only	285 (13.5%)	528 (3.9%)	3.87 (3.32–4.51)
Male	Adversity, then MH	43 (2.7%)	50 (0.5%)	5.80 (3.84–8.74)
	MH, then Adversity^a^	30 (1.9%)	30 (0.3%)	6.70 (4.03–11.14)
	Both simultaneously	40 (2.5%)	52 (0.5%)	5.17 (3.41–7.84)
	Adversity only	159 (9.9%)	812 (7.7%)	1.32 (1.11–1.58)
	MH only	190 (11.9%)	365 (3.5%)	3.76 (.13–4.52)
Female	Adversity, then MH	38 (7.5%)	14 (0.5%)	17.58 (9.45–32.70)
	MH, then Adversity^a^	20 (3.9%)	10 (0.3%)	12.49 (5.81–26.85)
	Both simultaneously	22 (4.3%)	20 (0.7%)	6.88 (3.72–12.70)
	Adversity only	42 (8.3%)	160 (5.2%)	1.63 (1.15–2.33)
	MH only	95 (18.7%)	163 (5.3%)	4.09 (3.11–5.37)

MH, mental health.

a. Frequencies rounded to nearest 10 to avoid disclosing small cell counts. Frequencies and odds ratios of individuals with admissions before age 18 coded for adverse events and/or mental health-related diagnoses, with the relative order of the first occurrence of each of the two types of events, by case status and sex. There were five possible combinations of events: adverse event first followed by mental health or vice versa, both happening simultaneously, or only adverse event or mental health alone. Adverse events were defined as in-patient hospital admissions meeting criteria for maltreatment or violence-related (MVR), care experience or no fixed abode, accidental poisonings, or the date of maternal bereavement; mental health events were defined as admissions meeting Clinical Classifications Software criteria, excluding admissions also meeting MVR or ‘care experience or no fixed abode’ criteria. The first occurrence of an adverse event and mental health event were derived separately, and the time elapsed was calculated for individuals who had both types of events. Events were considered simultaneous if they occurred within 28 days. Admissions coded for death were excluded. Denominators are the size of cohort with lifetime hospital records (*N* = 2106 for cases, *N* = 13 589 for controls).

Experiencing both adverse events and mental health admissions was associated with suicide more strongly, odds ratio_‘Adversity, then mental health’_ = 8.45 (95% CI, 6.07–11.77), odds ratio_‘mental health, then Adversity’_ = 8.24 (95% CI, 5.42–12.52), odds ratio_‘Both simultaneously’_ = 5.69 (95% CI, 4.04–8.02), than having only mental health admissions, odds ratio = 3.87 (95% CI, 3.32–4.51), or only adverse event admissions, odds ratio = 1.37 (95% CI, 1.17–1.61). This pattern was more pronounced with higher odds ratios in females.

### Ranking and dose-response relationship of adversity and mental health with suicide

**Fig. 2:**
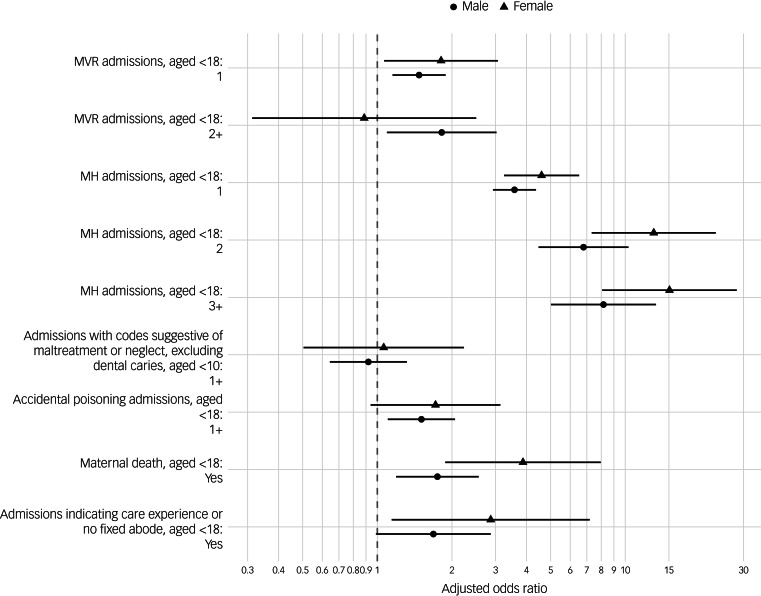
Maltreatment and violence-related (MVR), mental health-related (MH), adjusted odds ratio plot showing estimated effects of explanatory variable on case status (suicide) using conditional logistic regression (model 1), with estimates for male and female strata shown separately. Reference levels were having no respective admissions, or not having experienced maternal death. Horizontal line segments represent 95% CI.

A total of *N* = 12 136 males and *N* = 3559 females aged 0–18 were included in the regression analysis models 1 and 2, stratified by sex assigned at birth. Model 1 adjusted odds ratios (aOR), 95% CIs, and *P*-values for estimated effects of adverse events and mental health diagnoses on outcome are shown in Table 5 and visualised in Fig. 2; Model 2 aORs are shown in Table 6. Univariate statistics for Models 1 and 2 are shown in Supplementary Tables 11 and 12, respectively.

**Table 5:** Model 1: adjusted odds ratios of adverse events from conditional logistic regression with case status outcome, stratified by sex

	Stratum	Male			Female		
Adverse events	Level	aOR	95% CI	*P*	aOR	95% CI	*P*
MVR admissions, aged <18	1	1.47	1.15–1.88	0.002	1.81	1.07–3.07	0.028
	2+	1.82	1.09–3.02	0.021	0.89	0.31–2.51	0.819
MH admissions, aged <18	1	3.57	2.93–4.36	<0.0001	4.60	3.25–6.51	<0.0001
	2	6.79	4.47–10.30	<0.0001	13.03	7.33–23.17	<0.0001
	3+	8.17	5.02–13.29	<0.0001	15.08	8.07–28.17	<0.0001
Admissions with codes suggestive of maltreatment or neglect, excluding dental caries, aged <10	1+	0.92	0.64–1.32	0.649	1.06	0.50–2.23	0.875
Accidental poisoning admissions, aged <18	1+	1.51	1.10–2.06	0.010	1.72	0.94–3.14	0.079
Maternal death, aged <18	Yes	1.75	1.19–2.56	0.0040	3.87	1.88–7.97	0.000242
Admissions indicating care experience or no fixed abode, aged <18	Yes	1.68	0.99–2.87	0.056	2.87	1.14–7.19	0.025

aOR, adjusted odds ratio; MVR, maltreatment and violence-related; MH, mental health; estimates of adjusted odds ratios and confidence intervals in conditional logistic regression model of adversities with case status as outcome. Odds ratios were computed by exponentiating the multiple regression β coefficients. The reference level for all adversities was having no admissions or no record of maternal death before age 18. *N* = 12 136 individuals were included in the male stratum, and *N* = 3559 individuals in the female stratum.

**Table 6:** Model 2: adjusted odds ratios of number of types of adversity and number of mental health-related admissions from conditional logistic regression with case status outcome, stratified by sex

Stratum	Male			Female		
Explanatory variable	aOR	95% CI	*P*	aOR	95% CI	*P*
Number of types of adversity before age 18	1.90	1.64–2.21	<0.0001	2.65	1.94–3.62	<0.0001
Number of MH admissions before age 18	2.06	1.81–2.34	<0.0001	1.78	1.50–2.10	<0.0001
Interaction term: number of types of adversity × number of MH admissions before age 18	0.76	0.66–0.86	<0.0001	0.95	0.76–1.16	0.620

aOR, adjusted odds ratio; MH, mental health; estimates of adjusted odds ratios and confidence intervals in conditional logistic regression model with case status as outcome. Odds ratios were computed by exponentiating the multiple regression β coefficients. Individuals were coded as having experienced between zero and four types of adversity, defined as: maltreatment and violence-related (MVR) admissions, accidental poisoning admissions, maternal bereavement, and admissions indicative of care experience or no fixed abode. Mental health admissions were defined as admissions meeting Clinical Classifications Software criteria before age 18. *N* = 12 136 individuals were included in the male stratum and *N* = 3559 individuals in the female stratum.

In Model 1, after adjustment, one MVR admission (compared with none) was statistically significantly associated with outcome of suicide in both sexes, aOR_male_ = 1.47 (95% CI, 1.15–1.88) and aOR_female_ = 1.81 (95% CI, 1.07–3.07). Two or more MVR admissions were significantly associated with suicide in the male stratum, aOR_male_ = 1.82 (95% CI, 1.09–3.02) but not the female stratum, aOR_female_ = 0.89 (95% CI, 0.31–2.51). A single mental health admission was strongly associated with suicide in both sexes, aOR_male_ = 3.56 (95% CI, 2.93–4.36) and aOR_female_ = 4.60 (95% CI, 3.25–6.51), as was having two mental health admissions, aOR_male_ = 6.79 (95% CI, 4.47–10.30) and aOR_female_ = 13.03 (95% CI, 7.33–23.17), or three or more mental health admissions, aOR_male_ = 8.17 (95% CI, 5.02–13.29) and aOR_female_ = 15.08 (95% CI, 8.07–28.17).

Statistically significant associations with suicide were found in both strata for maternal bereavement, aOR_male_ = 1.75 (95% CI, 1.19–2.56) and aOR_female_ = 3.87 (95% CI, 1.88–7.97); Accidental poisoning was associated with suicide in males, aOR_male_ = 1.51 (95% CI, 1.10–2.06, *P* = 0.010) but not females, aOR_female_ = 1.72 (95% CI, 0.94–3.14, *P* = 0.079). Admissions indicative of ‘care experience’ or having no fixed abode were significantly associated with suicide in females, aOR_female_ = 2.87 (95% CI, 1.14–7.19) but not in males, aOR_male_ = 1.68 (95% CI, 0.99–2.87).

Model 2 revealed a significant association with suicide, for both sexes, for both the number of types of adversity, aOR_male_ = 1.90 (95% CI, 1.64–2.21) and aOR_female_ = 2.65 (95% CI, 1.94–3.62), and for number of mental health admissions, aOR_male_ = 2.06 (95% CI, 1.81–2.34) and aOR_female_ = 1.78 (95% CI, 1.50–2.10). In addition, there was a significant interaction between number of types of adversity and number of mental health admissions in the male stratum, aOR_male_ = 0.76 (95% CI, 0.66–0.86). The interaction in the female stratum was non-significant, aOR_female_ = 0.95 (95% CI, 0.76–1.16). A frequency table of the overlap between adversity and mental health admissions is in Supplementary Table 13.

## Discussion

### Main findings

Our lifespan comparison of cases and controls revealed statistically significant associations between adversity and mental health diagnoses in child and adolescent in-patient hospital records and later suicide as young adults. We ranked the relative impact of types of adverse events and mental health diagnoses: when the effects of childhood adversities and mental health diagnoses were adjusted for in a conditional logistic regression (Model 1), the strongest association in both sexes was for mental health admissions, followed in males by two or more MVR admissions, maternal bereavement, accidental poisoning and one MVR admission, and in females by maternal bereavement, admissions indicating care experience or no fixed abode, and one MVR admission.

A dose-response effect was evident for mental health admissions with odds increasing with the number of admissions from one to three or more in both sexes; for MVR admissions, the evidence for a dose-response was mixed: in males, there was an increase from having one admission, aOR_male_ = 1.47 (95% CI, 1.15–1.88), to having two or more, aOR_male_ = 1.82 (95% CI, 1.09–3.02), but not in females, where there was no significant effect of having two or more MVR admissions.

When we investigated the effect of the number of types of adversity and of mental health admissions in Model 2, each adversity type multiplied the odds of suicide by 1.9 in males, and 2.65 in females, and each mental health admission multiplied the odds of suicide by 2.06 in males and 1.78 in females. In males there was a significant interaction factor of 0.76 for individuals who experienced both adversity and mental health admissions, meaning that the combined effect of experiencing both adversity and mental health admissions was less than the sum of the individual effects (but still greater than either of the individual effects).

A rudimentary analysis of the relative occurrence of childhood adversities versus mental health admissions showed that individuals experiencing adversity followed by mental health admissions were somewhat more frequent than those experiencing both within 28 days, or with mental health admission first followed by adversity. This finding together with the negative interaction (Model 2) between the number of types of adversity and the number of mental health admissions in males, points towards a potential mediation effect, i.e. mental health admissions being protective against suicide in individuals who experienced adversity.

Presentations coded for adverse events and mental health admissions were rare in childhood (age <10) and were more common in adolescence (ages 10–17). We found hospital data before age 10 were sparse and could not identify sub-groups of children with increased vulnerability to later suicide, so the regression analysis used admissions aged 0–18.

A supplementary logistic regression model investigating ‘subcategories’ of MVR and mental health admissions (see Supplementary Tables 14 and 15) revealed that the type of MVR most strongly associated with suicide was ‘assault or maltreatment’, and the type of mental health admission most strongly associated with suicide was self-harm, followed by alcohol-related admissions in females, and non-alcohol-nor-self-harm admissions (NANSHR) in males.

### Comparisons with other studies

We found a 3:1 ratio of male to female deaths by suicide, but females accrued 31.5% of all hospitalisations, or 36.3% of psychiatric admissions; a similar study in Wales also found that females who died by suicide had more contacts than males across primary and secondary care settings.^17^ In controls, females accrued 23.0% of hospitalisations, or 28.6% of psychiatric admissions.

A previous study of suicide in Scotland between 1981 and 2009 of persons of all ages^28^ found that men died earlier than women, but in the younger cohort as per this study, men died approximately six months later on average (see Table 1 for details).

Sexual assault was recorded very rarely, despite its strong association with suicide found in other studies.^9^ Most adversities and mental health diagnoses were recorded in general hospital rather than during psychiatric admissions, though cases accrued more psychiatric admissions than controls (11.6% *v*. 1.4% of all admissions).

Codes suggestive of maltreatment or neglect were not associated with suicide after adjusting for other adversities. Unlike the original study,^25^ we additionally omitted the use of dental caries diagnoses as suggestive of neglect (see Supplementary file for details) and also found no effect after adjustment.

We found previously identified strong associations between suicide and mental health diagnoses, alcohol misuse, drug misuse and self-harm;^17,36,37^ these are potentially explained by the severity of such contacts in secondary care.^17^ Future studies could assess measures of severity of presentations, e.g. by including length of stay in hospital.

Of the adversities studied, MVR adversity was relatively common among adolescents, with 7.7% of cases having experienced their first episode aged 10–17. In male adolescents this was mainly accounted for by assaults. The experience of violence and its association with suicide should be given more priority in both research and policy, as has been highlighted elsewhere.^21^ Our study was not able to detect when victims of violence were also perpetrators; victimisation has been associated with later violence and psychological distress,^38^ and both victimisation and perpetration have been associated with suicide.^39^

### Strengths and limitations

We analysed up to 36 years of lifespan hospital admissions due to availability of data from 1981. Controls were defined as individuals alive at the time of their matched cases’ death, so it is possible that controls included some individuals who died by suicide after the study period.

Individuals with at least one hospitalisation prior to death, and their matched controls, were used as the denominator in computing odds ratios, to reflect hospital admission as a potential focus for suicide intervention. This was used instead of the total number of cases and controls identified, the consequence being that our reported odds ratios were lower but more easily interpretable. The alternative would have resulted in even higher odds ratios for childhood adversity due to the differing proportions of cases versus controls with any lifetime hospitalisations (approximately 85 and 55% for cases and controls, respectively).

Patient identifiers were extracted from patient registrations, which is considered the most complete register of persons in Scotland^40^; hospital records had high degree of completeness (between 93–99% during 2004–2006 and 2010–2011 assessments,^41^ and 99% in 2017^42^), so it is reasonable to assume that an individual with no hospitalisations genuinely had none, rather than representing a case of failed linkage. The difference in hospitalisations between cases and controls is therefore unlikely to represent a form of selection bias.

Despite our attempt at producing a highly sensitive set of criteria for adverse experiences recorded in hospitalisations, we found a low rate of adverse experiences, consistent with other studies^23,24^ and a consensus view that this is because of under-recording: only a subset of adverse experiences are recorded in hospital, reflecting relatively severe physical manifestations of adversity (‘tip of the iceberg’). This is compounded by the lack of diagnostic categories for possible or suspected maltreatment or neglect; clinicians may lack training or experience, have concerns about services being able to respond adequately, have concerns about clinician-family interactions or think recording maltreatment would do more harm than good.^23,43^ Similarly, we only identified a small number of adversities before age 10, which reflects how and what adversities are recorded in in-patient records rather than a true rate of adversities for this age group, which are likely to be far higher. In-patient records likely captured only relatively severe mental health presentations and presentations at a relatively higher age. Incorporating primary care data would provide an opportunity to include earlier and less severe presentations than those seen in hospital, potentially with different patterns than the relatively severe presentations studied here. National out-patient and primary care data were not available at the same level of quality or for the duration of the study period, but could be considered in future studies to capture less severe recorded adversity, depending on availability.

Because of the scarcity of adversity records, we collapsed all admissions before age 18 into a single category, precluding an analysis of the timing of adversity or mental health admission. Future studies could investigate this using a longitudinal approach, or e.g. time-to-event analysis.

Self-harm coding in ICD-9 and ICD-10 cannot distinguish cases of suicidal intent from other kinds of self-harm. Suicidal ideation can be coded (R45.8 in ICD-10) but was practically never used in the extracted data so we could not distinguish suicidal behaviours from thoughts. Self-harm coding was therefore very crude and encompassed a range of behaviours that may show different risk profiles when disentangled.^44^

Controls were matched to cases on death certificate postcode to control for (postcode-derived) individual level deprivation and rurality. Consequently, we controlled for ecological fallacy at the time of death but were unable to estimate effects of deprivation or rurality.

Our analysis of the relative order of occurrence of first presentations for childhood adversity and mental health admissions showed that adverse event admissions followed by mental health admissions were somewhat more frequent than vice versa. Together with the negative interaction between number of types of adversity and number of mental health admissions in males, they point towards a mediation effect between the two, though the present analysis could not determine the exact nature of the effect. Specifically, individuals with more than one type of adversity, and individuals with both adversity and mental health admissions, were rare. Further studies could improve sensitivity by combining additional data sources, such as community health data, with further mediation or interaction analyses in a causal framework (e.g.^45^).

### Implications for research

Consensus among data researchers^23–25,46^ is that adversities are underreported in administrative data, and this was also observed in this hospital admission data study. Previous research improved on this by cross-referencing data against specialist data-sets such as the Trauma Audit & Research Network (TARN) data collection,^23^ or child protection services’ data^25^ including information on social care involvement. Further research should explore the possibility of including social work records, and data on ‘looked after children’ which our study (though using very sparse data) indicated as a vulnerable group. Community health data also likely contains presentations for adverse events, as well as mild/moderate severity mental ill health presentations, and would be an important addition to data linkage.

The impact of adversity on school performance and exclusions, and their associations with suicide, can be studied by including education data.^47^ Potential also exists for further exploring maternal records, including MVR with mental health admissions. Finally, paternal record linkage to birth records and maternal records described in our protocol^14^ would allow a move towards a more complete picture of intergenerational adversity and its effects.

### Implications for policy and practice

Healthcare providers should prioritise suicide prevention activity in adolescents admitted as in-patients or day cases with childhood adversity (coded as violence and maltreatment) *and* mental health presentations in in-patient hospital records (general and/or psychiatric hospitals), as both were associated with greater odds of subsequent young person suicide. This demonstrates the need for good information sharing between general and psychiatric hospital systems, previously reported elsewhere.^28^

The strongest association of suicide in young people for a single type of adversity was self-harm in adolescence. There is a window of opportunity for healthcare practitioners to identify and flag potential ‘at-risk’ adolescents to engage in early intervention, and preventive and supportive actions to prevent future suicidal acts. Attention by healthcare professionals in general hospital settings should include greater scrutiny of poisonings coded as accidental, as these were associated with later suicide and likely represented a mixture of intentional and accidental poisonings.

## Supporting information

Dougall et al. supplementary material 1 Dougall et al. supplementary material

Dougall et al. supplementary material 2 Dougall et al. supplementary material

## Data Availability

The raw data used in this study are available on application from Public Health Scotland via the Public Benefit and Privacy Panel for Health and Social Care (HSC-PBPP, https://www.informationgovernance.scot.nhs.uk/pbpphsc/). Derived data that support the findings of this study are available on request from the corresponding author, J.S.
