# Supplementary material for: Childhood adversity and mental health admission patterns prior to young person suicide (CHASE): a case-control 36 year linked hospital data study, Scotland UK 1981–2017
Source: BJPsych Open. 2024 Jun 3;10(4):e124. doi: 10.1192/bjo.2024.69 (PMC11363076; doi:10.1192/bjo.2024.69)
Supplement: Dougall et al. supplementary material 1 — Dougall et al. supplementary material [file S2056472424000693sup001.docx]

Childhood adversity and mental health admission patterns prior to young person suicide (CHASE): a case-control 36 year linked hospital data study, Scotland UK 1981-2017

Supplementary materials

[Methodology 2](#_Toc152862560)

[Definition of ‘Probable suicide’ in Scotland 2](#_Toc152862561)

[Equivalent chapters between ICD-9 and ICD-10 2](#_Toc152862562)

[Supplementary table 1: Equivalent chapters between ICD versions 9 and 10 2](#_Toc152862563)

[Codes added to CCS classification & accidental poisoning category 3](#_Toc152862564)

[Supplementary table 2: Code ranges used to determine intentional self-injury, undetermined intent, and accidental poisoning codes 3](#_Toc152862565)

[Case-control matching algorithm 3](#_Toc152862566)

[Supplementary table 3: Case-control matching precision, matching by birth year 4](#_Toc152862567)

[Supplementary table 4: Case-control matching precision, matching by geography (postcode) 4](#_Toc152862568)

[Deprivation variables over years 4](#_Toc152862569)

[Supplementary table 5: Carstairs index versions used depending on year of record 4](#_Toc152862570)

[Supplementary table 6: SIMD versions used depending on year of record 4](#_Toc152862571)

[Codes used for care-experienced children and ‘no fixed abode’ 5](#_Toc152862572)

[Supplementary table 7: admission transfer from/discharge transfer to codes used to classify individuals with care experience or ‘no fixed abode’ 5](#_Toc152862573)

[Descriptive statistics 6](#_Toc152862574)

[Supplementary table 8: Distribution of ICD codes in hospital admissions under age 18 by ICD chapter, sex, and case status 6](#_Toc152862575)

[Supplementary Table 9: Mental health diagnosis frequency in hospitalisations before age 18, by sex and case status 7](#_Toc152862576)

[Supplementary Table 10: Frequency of poisoning codes by intent, sex, age at first admission, and case status 7](#_Toc152862577)

[Supplementary Table 11: Model 1 univariate descriptive statistics of adverse events & mental health-related admissions used as explanatory variables (conditional logistic regression) 8](#_Toc152862578)

[Supplementary Table 12: Model 2 univariate descriptive statistics of types of adverse events and number of mental health-related admissions before age 18 9](#_Toc152862579)

[Supplementary Table 13: Frequency of individuals experiencing combination of one or more types of adverse events and one or more mental health-related admission 9](#_Toc152862580)

[Supplementary Table 14: Univariate descriptive statistics of ‘subcategorised’ MVR admissions and MH admissions 10](#_Toc152862581)

[Supplementary Table 15: adjusted odds ratios of adverse events from conditional logistic regression using ‘subcategorised’ MVR and MH admissions, with case status outcome, stratified by sex 11](#_Toc152862582)

[Supplementary Table 16: adjusted odds ratios of adverse events from conditional logistic regression using ‘subcategorised’ MVR and MH admissions, with case status outcome, stratified by sex, excluding self-harm as explanatory variable 12](#_Toc152862583)

[Supplementary Figure 1: adjusted odds ratios with 95% confidence intervals from conditional logistic models using ‘subcategorised’ MVR and MH admissions, comparing models with or without self-harm as explanatory variable 13](#_Toc152862584)

[Note on codes suggestive of maltreatment or neglect 14](#_Toc152862585)

[Details of conditional logistic regression 14](#_Toc152862586)

[Supplementary Table 17: Generalised Variance Inflation Factor (GVIF) 15](#_Toc152862587)

[Controlling for geographical variables 15](#_Toc152862588)

[A note on (non-)overlapping adversity categories in logistic regression model 15](#_Toc152862589)

[Note on variable selection 15](#_Toc152862590)

[References 16](#_Toc152862591)

# Methodology

## Definition of ‘Probable suicide’ in Scotland

In Scotland, deaths by suicide are reported in terms of ‘probable suicide’, which includes deaths due to events of undetermined intent, the majority of which are assumed to have been suicides. In terms of ICD codes, this includes intentional self-harm (ICD-9 codes E950-E959; ICD-10 codes X60-X84 plus Y87.0) and events of undetermined intent (ICD-9 codes E980-E989; ICD-10 codes Y10-Y34 plus Y87.2)^1^. Note that this differs from the approach taken by the Office for National Statistics (ONS) in England & Wales, where probable suicides are based on codes for intentional self-harm for ages 10 or above and undetermined intent for ages 15 or above^2^. In the present study, either code was included from age 10 or above.

## Equivalent chapters between ICD-9 and ICD-10

The below table was used for computing the frequency tables of diagnoses by ICD chapters over both ICD-9 and ICD-10 versions. ICD-10 Chapter XXII – Codes for special purposes doesn’t have an ICD-9 equivalent but was not used in any of the extracted records.

Supplementary table 1: Equivalent chapters between ICD versions 9 and 10

| **ICD-9** | | **ICD-10** | |
| --- | --- | --- | --- |
| 1 | I | 1 | I |
| 10 | X | 14 | XIV |
| 11 | XI | 15 | XV |
| 12 | XII | 12 | XII |
| 13 | XIII | 13 | XIII |
| 14 | XIV | 17 | XVII |
| 15 | XV | 16 | XVI |
| 16 | XVI | 18 | XVIII |
| 17 | XVII | 19 | XIX |
| 2 | II | 2 | II |
| 3 | III | 4 | IV |
| 4 | IV | 3 | III |
| 5 | V | 5 | V |
| 6 | VI | 6,7,8 | VI,VII,VIII |
| 7 | VII | 9 | IX |
| 8 | VIII | 10 | X |
| 9 | IX | 11 | XI |
| E-codes | | 20 | XX |
| V-codes | | 21 | XXI |
| NA | | 22 | XXII |

Footnotes: NA: not applicable

## Codes added to CCS classification & accidental poisoning category

After data were extracted from the Scottish Morbidity Record (SMR), we discovered several codes that were related to mental health but were missed in the initial cross-mapping from ICD-9-CM and ICD-10-CM codes to ICD-9 and ICD-10 to obtain a list of mental health-related Clinical Classifications Software (CCS) codes. This is likely due to a combination of an older revision of ICD-10 having been used for cross-mapping (as available from the UK Biobank data dictionary (<https://biobank.ctsu.ox.ac.uk/crystal/coding.cgi?id=19>), and codes used for clinical coding specific to Scotland. The missing codes were included in the CCS code list by prefix-matching on existing CCS codes. For example, the ICD-9 code 295.03, not originally included in CCS but found in the SMR data, was assumed to be a subtype of 295.0 (‘Schizophrenic psychosis, simple type’) and was therefore matched by code 295.0 as it is a prefix of 295.03. This also captured all codes where a filler ‘X’ character was used to denote a missing fifth character, for example. In addition, a range of codes in ICD-9 and ICD-10 (see Supplementary table 3) were used to include codes for intentional self-injury, events of undetermined intent and accidents. These two strategies captured all codes missed in the initial cross-mapping effort, and an updated list of CCS codes mapped to categories is available at <https://github.com/jsavn/CHASe-outside-safe-haven>.

Note that ICD-10 requires that poisonings where the intent cannot be distinguished between assault, accident, or self-harm, are coded using accidental poisoning codes, X40-X49, despite the available range Y10-Y19 specifying poisonings of undetermined intent:

*“[Y10-Y34] covers events where available information is insufficient to enable a medical or legal authority to make a distinction between accident, self-harm and assault. It includes self-inflicted injuries, but not poisoning, when not specified whether accidental or with intent to harm (X40-X49). Follow legal rulings when available.”* ^3^

On review, accidental poisoning codes (X40-X49, E850-E869) were relatively common so they were included as an adversity category separate to CCS codes. There were a very small number of poisonings of undetermined intent, and these were included in the CCS coding, under the ‘Undetermined intent’ heading.

Supplementary table 2: Code ranges used to determine intentional self-injury, undetermined intent, and accidental poisoning codes

| **Description** | **ICD version** | **Code range** | **Categorisation in present study** |
| --- | --- | --- | --- |
| Intentional self-injury | 10 | X60-X84 | CCS category 662: Suicide and intentional self-inflicted injury |
|  | 9 | E950-E959 |  |
| Undetermined intent | 10 | Y10-Y34 | CCS category 671: Events of undetermined intent |
|  | 9 | E980-E989 |  |
| Accidental poisoning | 10 | X40-X49 | Separate adversity category: Accidental poisoning |
|  | 9 | E850-E869 |  |

## Case-control matching algorithm

Controls were exactly matched to cases on sex and approximately on birth year and geography as recorded in cases’ death records. A 1:10 ratio was used for controls for a total of N=24,770. The following tables summarise the precision achieved in the matching, with nearly 99% of controls having the same birth year, and just over 98% of controls residing in the same postcode sector.

Controls were matched to cases by a trusted third party, the electronic Data Research and Innovation Service (eDRIS), which is part of Public Health Scotland (PHS), during data extraction^4^. The matching algorithm proceeded as follows: for each individual, a ‘pool’ of matching individuals were identified with the same sex, birth year within ±2 years, and geography (defined as Postcode sector – see Supplementary table 4 below). Where fewer than 10 individuals were available in the pool of potential matches, the geography criterion was relaxed (from Postcode sector to Postcode district, Postcode area, or all of Scotland, as required), until at least 10 individuals were available for matching.

Supplementary table 3: Case-control matching precision, matching by birth year

| **Birth year difference (case - control)** | **N** | **Proportion** |
| --- | --- | --- |
| -2 | 9 | 0.04% |
| -1 | 94 | 0.38% |
| 0 | 24,510 | 98.95% |
| 1 | 143 | 0.58% |
| 2 | 14 | 0.06% |

Supplementary table 4: Case-control matching precision, matching by geography (postcode)

| **Matching area** | **N** | **Proportion** | **Interpretation of area** |
| --- | --- | --- | --- |
| Postcode sector | 24,368 | 98.38% | Postcode Sector is postcode minus last two characters |
| Postcode district | 223 | 0.90% | Postcode District is postcode minus last three digits/characters |
| Postcode area | 89 | 0.36% | Postcode Area is first two characters of postcode (first character in Glasgow) |
| Scotland | 90 | 0.36% | Scotland-wide matching used for missing or invalid postcodes |

Footnotes: for cases, the postcode in the death record was matched to controls’ postcode in their CHI (Community Health Index) record at the time of the cases’ death.

## Deprivation variables over years

The data spanned 1981 to 2017 so the Carstairs Index and SIMD (Scottish Index of Multiple Deprivation) at all available years were computed from postcodes. For each record, the most appropriate Carstairs Index and SIMD were determined based on the date of death (Death records) or date of discharge (SMR01, SMR02, SMR04), according to guidance by ISD Scotland^5^:

Supplementary table 5: Carstairs index versions used depending on year of record

| **Carstairs index** | **Year of record** |
| --- | --- |
| 1981 | 1981-1985 |
| 1991 | 1986-1995 |
| 2001 | 1996-2005 |
| 2011 | 2006-2017 |

Supplementary table 6: SIMD versions used depending on year of record

| **SIMD index** | **Year of record** | **Note** |
| --- | --- | --- |
| 2004 | 1981-1995 | The earliest SIMD version is 2004 which is only advised for use with data from 1996 onwards; fewer than 0.5% of death records were prior to 1996, so the 2004 version was used for those also. |
| 2004 | 1996-2003 |  |
| 2006 | 2004-2006 |  |
| 2009 | 2007-2009 |  |
| 2012 | 2010-2013 |  |
| 2016 | 2014-2017 |  |

Footnotes: SIMD: Scottish Index of Multiple Deprivation

All deprivation variables were expressed as deciles and were recoded so that the value of 1 represented the most deprived decile^5^. Because of changes to postcodes over time it may not be possible to compute a Carstairs Index and SIMD for a particular record for all the available deprivation variable years. For this reason, we used an algorithm to choose either the deprivation variable as determined in the above tables where available, or the closest more recent available deprivation variable (e.g. for a 2003 record, we would use the 2004 SIMD index if available, or the 2006 index if 2004 was not available, or the 2009 index if neither 2004 nor 2006 were available, and so on). However, in all cases with any available deprivation variables, the appropriate variable according to the above tables was also available.

## Codes used for care-experienced children and ‘no fixed abode’

*Admission transfer from* and *Discharge transfer to* codes were used to classify admissions as coming from or being discharged to care-related institutions and these were used to identify individuals with experience of care or individuals at risk of experiencing homelessness (‘no fixed abode’). SMR01 and SMR04 variables ADMISSION_TRANSFER_FROM and DISCHARGE_TRANSFER_TO were available in data from April 1996 onward. Additionally, the variable OLD_ADMITTED_FROM was available for SMR01 data prior to April 1996. The SMR code dictionaries at <https://www.ndc.scot.nhs.uk/Data-Dictionary/SMR-Datasets/Episode-Management/Discharge-Transfer-To/> and <https://www.ndc.scot.nhs.uk/Data-Dictionary/SMR-Datasets/Episode-Management/Admission-Transfer-From/> were manually reviewed. Codes indicating care homes, residential institutions, and ‘unusual’ places of residence were included as indicative of care experience and were found to be relatively rare. ‘No fixed abode’ was also very rare, and so was combined with places indicative of care experience to form a single, place of residence-based adversity category.

Supplementary table 7: admission transfer from/discharge transfer to codes used to classify individuals with care experience or ‘no fixed abode’

| **Category** | **Code** |
| --- | --- |
| Care experience | 14 - Private Residence - (supported) |
|  | 18 - Private Residence - Other type (e.g.Foster Care) |
|  | 20 Place of Residence - Institution, no additional detail added |
|  | 25 Care home |
|  | 28 Place of Residence - Institution - other type |
|  | 29 Place of Residence - Institution - type not known |
|  | 33 - Legal establishment, including prison |
|  | 38 - Other type of temporary residence (includes hospital residences, hotel facilities) |
|  | 39 - Temporary place of residence - type not known |
| No fixed abode | 34 – No fixed abode |

# Descriptive statistics

Supplementary table 8: Distribution of ICD codes in hospital admissions under age 18 by ICD chapter, sex, and case status

|  |  | **Main diagnosis** | | | | **Any diagnosis** | | | |
| --- | --- | --- | --- | --- | --- | --- | --- | --- | --- |
|  |  | **Cases** | | **Controls** | | **Cases** | | **Controls** | |
| **ICD Chapter** |  | **Male** | **Female** | **Male** | **Female** | **Male** | **Female** | **Male** | **Female** |
| Hospitalisations before age 18 | N | 4,993 | 1,883 | 27,662 | 7,202 | 4,993 | 1,883 | 27,662 | 7,202 |
| Infectious and parasitic diseases | N (%) | 175 (3.5%) | 72 (3.8%) | 1,164 (4.2%) | 331 (4.6%) | 233 (4.7%) | 105 (5.6%) | 1,474 (5.3%) | 423 (5.9%) |
| Neoplasms |  | 153 (3.1%) | 50 (2.7%) | 805 (2.9%) | 236 (3.3%) | 178 (3.6%) | 52 (2.8%) | 871 (3.1%) | 272 (3.8%) |
| Endocrine, nutritional and metabolic diseases, and immunity disorders |  | 56 (1.1%) | 88 (4.7%) | 270 (1.0%) | 85 (1.2%) | 105 (2.1%) | 109 (5.8%) | 423 (1.5%) | 163 (2.3%) |
| Diseases of the blood and blood-forming organs |  | 76 (1.5%) | 10 (0.5%) | 509 (1.8%) | 56 (0.8%) | 116 (2.3%) | 17 (0.9%) | 682 (2.5%) | 79 (1.1%) |
| Diseases of the nervous system and the sense organs |  | 308 (6.2%) | 119 (6.3%) | 2,221 (8.0%) | 669 (9.3%) | 416 (8.3%) | 171 (9.1%) | 3122 (11.3%) | 845 (11.7%) |
| Mental disorders |  | 186 (3.7%) | 115 (6.1%) | 300 (1.1%) | 128 (1.8%) | 333 (6.7%) | 230 (12.2%) | 590 (2.1%) | 243 (3.4%) |
| Diseases of the circulatory system |  | 24 (0.5%) | 20 (1.1%) | 160 (0.6%) | 51 (0.7%) | 40 (0.8%) | 39 (2.1%) | 254 (0.9%) | 90 (1.2%) |
| Diseases of the respiratory system |  | 660 (13.2%) | 232 (12.3%) | 4,212 (15.2%) | 1145 (15.9%) | 849 (17.0%) | 295 (15.7%) | 5,120 (18.5%) | 1,375 (19.1%) |
| Diseases of the digestive system |  | 666 (13.3%) | 207 (11.0%) | 4,151 (15.0%) | 1156 (16.1%) | 745 (14.9%) | 239 (12.7%) | 4,527 (16.4%) | 1,254 (17.4%) |
| Diseases of the genitourinary system |  | 222 (4.4%) | 39 (2.1%) | 1,751 (6.3%) | 219 (3.0%) | 263 (5.3%) | 54 (2.9%) | 2,000 (7.2%) | 289 (4.0%) |
| Complications of pregnancy, childbirth, and the puerperium |  |  | 51 (2.7%) |  | 191 (2.7%) |  | 51 (2.7%) |  | 195 (2.7%) |
| Diseases of the skin and subcutaneous tissue |  | 112 (2.2%) | 30 (1.6%) | 751 (2.7%) | 201 (2.8%) | 141 (2.8%) | 43 (2.3%) | 934 (3.4%) | 236 (3.3%) |
| Diseases of the musculoskeletal system and connective tissue |  | 119 (2.4%) | 36 (1.9%) | 663 (2.4%) | 193 (2.7%) | 166 (3.3%) | 51 (2.7%) | 763 (2.8%) | 232 (3.2%) |
| Congenital anomalies |  | 257 (5.1%) | 18 (1.0%) | 1,931 (7.0%) | 369 (5.1%) | 338 (6.8%) | 36 (1.9%) | 2519 (9.1%) | 584 (8.1%) |
| Certain conditions originating in the perinatal period |  | 39 (0.8%) |  | 322 (1.2%) | 61 (0.8%) | 46 (0.9%) | 10 (0.5%) | 367 (1.3%) | 65 (0.9%) |
| Symptoms, signs, and ill-defined conditions |  | 464 (9.3%) | 211 (11.2%) | 2,448 (8.8%) | 937 (13.0%) | 645 (12.9%) | 297 (15.8%) | 3404 (12.3%) | 1,215 (16.9%) |
| Injury and poisoning |  | 1,297 (26.0%) | 517 (27.5%) | 4,947 (17.9%) | 922 (12.8%) | 1,347 (27.0%) | 531 (28.2%) | 5,126 (18.5%) | 963 (13.4%) |
| Supplementary classification of factors influencing health status and contact with health services |  | 179 (3.6%) | 60 (3.2%) | 1,057 (3.8%) | 252 (3.5%) | 563 (11.3%) | 254 (13.5%) | 2,625 (9.5%) | 762 (10.6%) |
| External causes of injury |  | NA | NA | NA | NA | 1,350 (27.0%) | 535 (28.4%) | 5,139 (18.6%) | 967 (13.4%) |

Footnotes: NA: Not applicable; Frequency of ICD codes by chapter in hospital admissions (physical health, SMR01, and psychiatric admissions, SMR04, combined), before age 18, for cases & controls, by whether they appeared in first position (‘Main diagnosis’) or any position. The denominator is the total number of hospital admissions before age 18 for cases & controls, respectively. Numbers ≤10 have been suppressed; external causes of injury cannot be used in Main diagnosis position. ICD-10 chapters were matched to ICD-9 chapters (see Supplementary table 2). Each admission has one main diagnosis and a variable number of secondary diagnoses, thus the numbers & percentages for ‘Any diagnosis’ do not add up to 100%. There was a large difference between frequencies of Mental disorders and Injury and poisoning chapter codes (and the accompanying External causes of injury codes) between cases and controls.

Supplementary Table 9: Mental health diagnosis frequency in hospitalisations before age 18, by sex and case status

|  |  | Cases | | | Controls | | | OR [95% CI] | | |
| --- | --- | --- | --- | --- | --- | --- | --- | --- | --- | --- |
| CCS Mental health-related category |  | Both | Male | Female | Both | Male | Female | Both | Male | Female |
| Anxiety disorders | N (%) | 27 (1.3%) | 10 (0.6%) | 17 (3.4%) | 62 (0.5%) | 39 (0.4%) | 23 (0.8%) | 2.83 [1.80-4.46] | 1.69 [0.84-3.40] | 4.57 [2.42-8.62] |
| Mood disorders |  | 53 (2.5%) | 22 (1.4%) | 31 (6.1%) | 32 (0.2%) | 13 (0.1%) | 19 (0.6%) | 10.94 [7.03-17.00] | 11.29 [5.68-22.46] | 10.40 [5.82-18.56] |
| Alcohol-related disorders |  | 192 (9.1%) | 132 (8.3%) | 60 (11.8%) | 283 (2.1%) | 225 (2.1%) | 58 (1.9%) | 4.72 [3.90-5.70] | 4.12 [3.30-5.15] | 6.93 [4.76-10.08] |
| Substance-related disorders |  | 84 (4.0%) | 56 (3.5%) | 28 (5.5%) | 52 (0.4%) | 42 (0.4%) | 10 (0.3%) | 10.81 [7.63-15.33] | 9.07 [6.06-13.58] | 17.78 [8.58-36.85] |
| Suicide and intentional self-inflicted injury |  | 295 (14.0%) | 145 (9.1%) | 150 (29.6%) | 207 (1.5%) | 103 (1.0%) | 104 (3.4%) | 10.53 [8.76-12.66] | 10.10 [7.80-13.08] | 11.91 [9.06-15.66] |
| Other |  | 149 (7.1%) | 92 (5.8%) | 57 (11.2%) | 259 (1.9%) | 192 (1.8%) | 67 (2.2%) | 3.92 [3.19-4.82] | 3.29 [2.55-4.24] | 5.64 [3.91-8.15] |

Footnotes: CCS: Clinical Classifications Software; OR: Odds ratio; CI: Confidence interval; Frequencies of individuals with given Mental health-related diagnoses before age 18. Diagnoses in any position were included; CCS categories are not mutually exclusive – the same individual can be counted in multiple categories. Note the higher proportion of all CCS categories for cases than controls. Due to small numbers, several CCS categories were combined into an 'Other' category: Adjustment disorders, Attention-deficit conduct and disruptive behaviour disorders, Delirium dementia and amnestic and other cognitive disorders, Developmental disorders, Disorders usually diagnosed in infancy childhood or adolescence, Impulse control disorders NEC, Personality disorders, Schizophrenia and other psychotic disorders, Screening and history of mental health and substance abuse codes, Miscellaneous mental health disorders, and the additional category for Events of undetermined intent.

Supplementary Table 10: Frequency of poisoning codes by intent, sex, age at first admission, and case status

|  |  |  | Cases | | | Controls | | | OR [95% CI] | | |
| --- | --- | --- | --- | --- | --- | --- | --- | --- | --- | --- | --- |
| External cause code (intent) for poisoning | Age |  | Both | Male | Female | Both | Male | Female | Both | Male | Female |
| Accidental or undetermined intent | <10 | N (%) | 84 (4.0%) | 60 (3.8%) | 24 (4.7%) | 374 (2.8%) | 298 (2.8%) | 76 (2.5%) | 1.47 [1.15-1.87] | 1.34 [1.01-1.78] | 1.95 [1.22-3.11] |
| Accidental or undetermined intent | >=10, <18 |  | 69 (3.3%) | 38 (2.4%) | 31 (6.1%) | 57 (0.4%) | 38 (0.4%) | 19 (0.6%) | 8.04 [5.64-11.46] | 6.73 [4.28-10.58] | 10.40 [5.82-18.56] |
| Intentional | >=10, <18 |  | 267 (12.7%) | 128 (8.0%) | 139 (27.4%) | 185 (1.4%) | 87 (0.8%) | 98 (3.2%) | 10.52 [8.67-12.77] | 10.45 [7.92-13.80] | 11.39 [8.60-15.07] |

Footnotes: OR: Odds ratio; CI: Confidence interval; Frequencies of individuals with an external cause code indicating poisoning (intentional E950-E952, X60-X69; accidental E850-E869, X40-X49; undetermined intent E980-E982, Y10-Y19), grouped by age at first admission and sex. ICD-10 requires poisonings with unclear intent (i.e. undetermined intent) to be coded as accidental, and undetermined intent codes were rare, so the two categories were combined. Both SMR01 and SMR04 records were included. Death-related hospital admissions were excluded. The denominators are the total number of individuals who died by suicide or controls who had at least one hospital record prior to death, grouped by sex. Index admission counted only (an individual was only counted at the earliest age for each external code for intent). For intentional poisonings only the 10-18 age group is shown due to small numbers and is practically identical with the <18 group. Poisoning-related external cause codes were used to identify poisonings rather than poisoning codes themselves because of higher coverage of hospital admissions: 0.57% of non-death-related admissions (1.76% of individuals) had a poisoning-related external cause code but no poisoning code, while only 0.06% of admissions (0.21% of individuals) had a poisoning code without an external cause code (with no way of inferring the external cause code).

Supplementary Table 11: Model 1 univariate descriptive statistics of adverse events & mental health-related admissions used as explanatory variables (conditional logistic regression)

|  | Stratum | Male | | | | Female | | | |
| --- | --- | --- | --- | --- | --- | --- | --- | --- | --- |
| Variable | Level | Cases N = 1,599 | Controls N = 10,537 | OR (95% CI) | p | Cases N = 507 | Controls N = 3,052 | OR (95% CI) | p |
| MVR admissions, aged <18 | 1 | 112 (7.0%) | 372 (3.5%) | 2.07 [ 1.65- 2.60] | <.0001 | 38 (7.5%) | 63 (2.1%) | 3.71 [ 2.39- 5.77] | <.0001 |
| MVR admissions, aged <18 | 2+ | 32 (2.0%) | 59 (0.6%) | 3.97 [ 2.52- 6.27] | <.0001 | 13 (2.6%) | 17 (0.6%) | 6.20 [ 2.83-13.62] | <.0001 |
| MH admissions, aged <18 | 1 | 198 (12%) | 398 (3.8%) | 3.96 [ 3.26- 4.80] | <.0001 | 80 (16%) | 150 (4.9%) | 5.19 [ 3.70- 7.27] | <.0001 |
| MH admissions, aged <18 | 2 | 56 (3.5%) | 57 (0.5%) | 8.03 [ 5.36-12.03] | <.0001 | 42 (8.3%) | 31 (1.0%) | 16.11 [ 9.33-27.83] | <.0001 |
| MH admissions, aged <18 | 3+ | 47 (2.9%) | 37 (0.4%) | 10.31 [ 6.43-16.51] | <.0001 | 58 (11%) | 21 (0.7%) | 22.49 [12.70-39.83] | <.0001 |
| Admissions with codes suggestive of maltreatment or neglect, excluding dental caries, aged <10 | 1+ | 59 (3.7%) | 307 (2.9%) | 1.27 [ 0.95- 1.70] | .108 | 21 (4.1%) | 67 (2.2%) | 1.87 [ 1.12- 3.12] | .017 |
| Accidental poisoning admissions, aged <18 | 1+ | 90 (5.6%) | 321 (3.0%) | 1.82 [ 1.42- 2.34] | <.0001 | 46 (9.1%) | 89 (2.9%) | 3.20 [ 2.17- 4.73] | <.0001 |
| Maternal death, aged <18 | Yes | 40 (2.5%) | 159 (1.5%) | 1.72 [ 1.20- 2.48] | .004 | 18 (3.6%) | 29 (1.0%) | 5.06 [ 2.65- 9.66] | <.0001 |
| Admissions indicating care experience or no fixed abode, aged <18 | Yes | 36 (2.3%) | 43 (0.4%) | 5.05 [ 3.18- 8.03] | <.0001 | 30 (5.9%) | 12 (0.4%) | 15.11 [ 7.03-32.44] | <.0001 |

Footnotes: MVR: Maltreatment or violence-related; MH: mental health; OR: odds ratio; Odds ratios shown for univariate conditional logistic regression with explanatory variable and outcome cases status. 95% OR confidence interval used profile likelihood.

Supplementary Table 12: Model 2 univariate descriptive statistics of types of adverse events and number of mental health-related admissions before age 18

|  | Stratum | Male | | | | Female | | | |
| --- | --- | --- | --- | --- | --- | --- | --- | --- | --- |
| Variable | Level | Cases N = 1,599 | Controls N = 10,537 | OR (95% CI) | p | Cases N = 507 | Controls N = 3,052 | OR (95% CI) | p |
| Number of types of adversity before age 18 | 1 | 229 (14%) | 854 (8.1%) | 1.89 [1.61-2.22] | <.0001 | 99 (20%) | 181 (5.9%) | 4.01 [4.01-5.35] | <.0001 |
| `` | 2+ | 39 (2.4%) | 47 (0.4%) | 6.31 [4.03-9.89] | <.0001 | 22 (4.3%) | 14 (0.5%) | 14.20 [6.35-31.73] | <.0001 |
| Number of MH admissions before age 18 | 1 | 198 (12%) | 398 (3.8%) | 3.96 [3.26-4.80] | <.0001 | 80 (16%) | 150 (4.9%) | 5.19 [3.70-7.27] | <.0001 |
| `` | 2 | 56 (3.5%) | 57 (0.5%) | 8.03 [5.36-12.03] | <.0001 | 42 (8.3%) | 31 (1.0%) | 16.11 [9.33-27.83] | <.0001 |
| `` | 3+ | 47 (2.9%) | 37 (0.4%) | 10.31 [6.43-16.51] | <.0001 | 58 (11%) | 21 (0.7%) | 22.49 [12.7-39.83] | <.0001 |

Footnotes: MH: mental health; OR: odds ratio; Odds ratios shown for univariate conditional logistic regression with explanatory variable and outcome cases status. 95% OR confidence interval used profile likelihood. Individuals were coded as having experienced between zero and four types of adversity, defined as: maltreatment and violence-related (MVR) admissions, accidental poisoning admissions, maternal bereavement, and admissions indicative of care experience or no fixed abode. MH admissions were defined as admissions meeting CCS criteria before age 18.

Supplementary Table 13: Frequency of individuals experiencing combination of one or more types of adverse events and one or more mental health-related admission

| *Sex* | Male | | Female | |
| --- | --- | --- | --- | --- |
| *Combination of adverse events and MH admissions N (%)* | Case | Control | Case | Control |
| No adverse events and No MH admissions | 1,145 (71.6%) | 9,280 (88.1%) | 292 (57.6%) | 2,699 (88.4%) |
| No adverse events and ≥1 MH admissions | 186 (11.6%) | 356 (3.4%) | 94 (18.5%) | 158 (5.2%) |
| ≥1 types of adverse event and No MH admissions | 153 (9.6%) | 765 (7.3%) | 35 (6.9%) | 151 (4.9%) |
| ≥1 types of adverse event and ≥1 MH admissions | 115 (7.2%) | 136 (1.3%) | 86 (17.0%) | 44 (1.4%) |

Footnotes: MH: mental health; Individuals were coded as having experienced between zero and four types of adversity, defined as: maltreatment and violence-related (MVR) admissions, accidental poisoning admissions, maternal bereavement, and admissions indicative of care experience or no fixed abode. MH admissions were defined as admissions meeting CCS criteria before age 18.

Supplementary Table 14: Univariate descriptive statistics of ‘subcategorised’ MVR admissions and MH admissions

|  | Stratum | Male | | | | Female | | | |
| --- | --- | --- | --- | --- | --- | --- | --- | --- | --- |
| Variable | Level | Cases N = 1,599 | Controls N = 10,537 | OR (95% CI) | p | Cases N = 507 | Controls N = 3,052 | OR (95% CI) | p |
| MVR admissions: assault or maltreatment, aged <18 | 1+ | 111 (6.9%) | 282 (2.7%) | 2.70 [ 2.13- 3.42] | <.0001 | 14 (2.8%) | 14 (0.5%) | 6.23 [ 2.79-13.93] | <.0001 |
| MVR admissions: adverse soc. circumstances or undetermined intent, aged <18 | 1 | 44 (2.8%) | 159 (1.5%) | 1.92 [ 1.35- 2.72] | .000245 | 30 (5.9%) | 54 (1.8%) | 3.25 [ 2.01- 5.25] | <.0001 |
| `` | 2+ | 13 (0.8%) | 30 (0.3%) | 3.33 [ 1.67- 6.64] | .000622 | 10 (2.0%) | 15 (0.5%) | 5.12 [ 2.17-12.03] | .000184 |
| MH admissions: self harm, aged <18 | 1+ | 145 (9.1%) | 103 (1.0%) | 10.29 [ 7.77-13.64] | <.0001 | 150 (30%) | 104 (3.4%) | 12.90 [ 9.33-17.82] | <.0001 |
| MH admissions: alcohol-related, aged <18 | 1+ | 132 (8.3%) | 225 (2.1%) | 4.19 [ 3.31- 5.31] | <.0001 | 60 (12%) | 58 (1.9%) | 7.68 [ 5.05-11.67] | <.0001 |
| MH admissions: neither self harm nor alcohol-related, aged <18 | 1 | 90 (5.6%) | 164 (1.6%) | 3.91 [ 2.97- 5.16] | <.0001 | 44 (8.7%) | 63 (2.1%) | 4.91 [ 3.19- 7.55] | <.0001 |
| `` | 2+ | 37 (2.3%) | 57 (0.5%) | 4.89 [ 3.15- 7.59] | <.0001 | 40 (7.9%) | 29 (1.0%) | 9.76 [ 5.62-16.98] | <.0001 |

Footnotes: MVR: Maltreatment or violence-related; MH: mental health; OR: odds ratio; Odds ratios shown for univariate conditional logistic regression with explanatory variable and outcome cases status. 95% OR confidence interval used profile likelihood. See Supplementary table 11 above for univariate descriptives of Admissions suggestive of maltreatment or neglect, Accidental poisoning, Maternal death, and Admissions indicating care experience or no fixed abode.

Supplementary Table 15: adjusted odds ratios of adverse events from conditional logistic regression using ‘subcategorised’ MVR and MH admissions, with case status outcome, stratified by sex

|  | Stratum | Male | | | Female | | |
| --- | --- | --- | --- | --- | --- | --- | --- |
| Adverse events | Level | aOR | 95% CI | p | aOR | 95% CI | p |
| MVR admissions: assault or maltreatment, aged <18 | 1+ | 1.91 | 1.47-2.50 | <.0001 | 3.83 | 1.41-10.34 | .0080 |
| MVR admissions: adverse soc. circumstances or undetermined intent, aged <18 | 1 | 1.01 | 0.68-1.51 | .95 | 1.58 | 0.87- 2.88 | .13 |
| `` | 2+ | 1.00 | 0.43-2.30 | 1 | 0.74 | 0.23- 2.37 | .61 |
| MH admissions: self-harm, aged <18 | 1+ | 6.67 | 4.93-9.02 | <.0001 | 7.31 | 5.03-10.61 | <.0001 |
| MH admissions: alcohol-related, aged <18 | 1+ | 2.45 | 1.87-3.20 | <.0001 | 2.33 | 1.37- 3.94 | .0020 |
| MH admissions: neither self-harm nor alcohol-related (NANSHR), aged <18 | 1 | 2.18 | 1.60-2.98 | <.0001 | 1.63 | 0.94- 2.84 | .082 |
| `` | 2+ | 2.67 | 1.61-4.44 | .00015 | 2.27 | 1.09- 4.73 | .029 |
| Admissions with codes suggestive of maltreatment or neglect, excluding dental caries, aged <10 | 1+ | 0.90 | 0.63-1.30 | .58 | 0.96 | 0.45- 2.05 | .91 |
| Accidental poisoning admissions, aged <18 | 1+ | 1.53 | 1.11-2.10 | .010 | 1.80 | 0.98- 3.30 | .056 |
| Maternal death, aged <18 | Yes | 1.70 | 1.15-2.51 | .0070 | 3.87 | 1.85- 8.06 | .00031 |
| Admissions indicating care experience or no fixed abode, aged <18 | Yes | 1.67 | 0.96-2.92 | .072 | 2.37 | 0.92- 6.13 | .074 |

Footnotes: aOR: Adjusted odds ratio; CI: Confidence interval; MVR: Maltreatment and violence-related; MH: Mental health; Estimates of adjusted odds ratios & confidence intervals in conditional logistic regression model of adversities with case status as outcome. Odds ratios were computed by exponentiating the multiple regression β coefficients. The reference level for all adversities was having no admissions or no record of maternal death before age 18. N=12,136 individuals were included in the male stratum and N=3,559 individuals in the female stratum.

Supplementary Table 16: adjusted odds ratios of adverse events from conditional logistic regression using ‘subcategorised’ MVR and MH admissions, with case status outcome, stratified by sex, excluding self-harm as explanatory variable

|  |  | Male | | Female | |
| --- | --- | --- | --- | --- | --- |
| Adverse events | Level | aOR | 95% CI | aOR | 95% CI |
| MVR admissions: assault or maltreatment, aged <18 | 1+ | 1.92 | 1.48-2.49 | 4.55 | 1.82-11.37 |
| MVR admissions: adverse soc. circumstances or undetermined intent, aged <18 | 1 | 1.17 | 0.80-1.71 | 1.94 | 1.11- 3.40 |
| `` | 2+ | 1.32 | 0.61-2.83 | 1.3 | 0.42- 4.02 |
| MH admissions: self harm, aged <18 | 1+ | NA | NA | NA | NA |
| MH admissions: alcohol-related, aged <18 | 1+ | 2.95 | 2.29-3.80 | 4.6 | 2.86- 7.41 |
| MH admissions: neither self harm nor alcohol-related, aged <18 | 1 | 2.8 | 2.09-3.77 | 2.99 | 1.82- 4.93 |
| `` | 2+ | 3.7 | 2.32-5.91 | 4.65 | 2.47- 8.76 |
| Accidental poisoning admissions, aged <18 | 1+ | 1.51 | 1.17-1.97 | 2.15 | 1.39- 3.33 |
| Maternal death, aged <18 | TRUE | 1.65 | 1.13-2.42 | 3.86 | 1.92- 7.76 |
| Admissions indicating care experience or no fixed abode, aged <18 | TRUE | 2.25 | 1.33-3.78 | 6.29 | 2.55-15.50 |
| MVR admissions: assault or maltreatment, aged <18 | 1+ | 1.92 | 1.48-2.49 | 4.55 | 1.82-11.37 |

Footnotes: aOR: Adjusted odds ratio; CI: Confidence interval; MVR: Maltreatment and violence-related; MH: Mental health; NA: Not applicable; Estimates of adjusted odds ratios & confidence intervals in conditional logistic regression model of adversities with case status as outcome. Odds ratios were computed by exponentiating the multiple regression β coefficients. The reference level for all adversities was having no admissions or no record of maternal death before age 18. N=12,136 individuals were included in the male stratum and N=3,559 individuals in the female stratum.

Supplementary Figure 1: adjusted odds ratios with 95% confidence intervals from conditional logistic models using ‘subcategorised’ MVR and MH admissions, comparing models with or without self-harm as explanatory variable


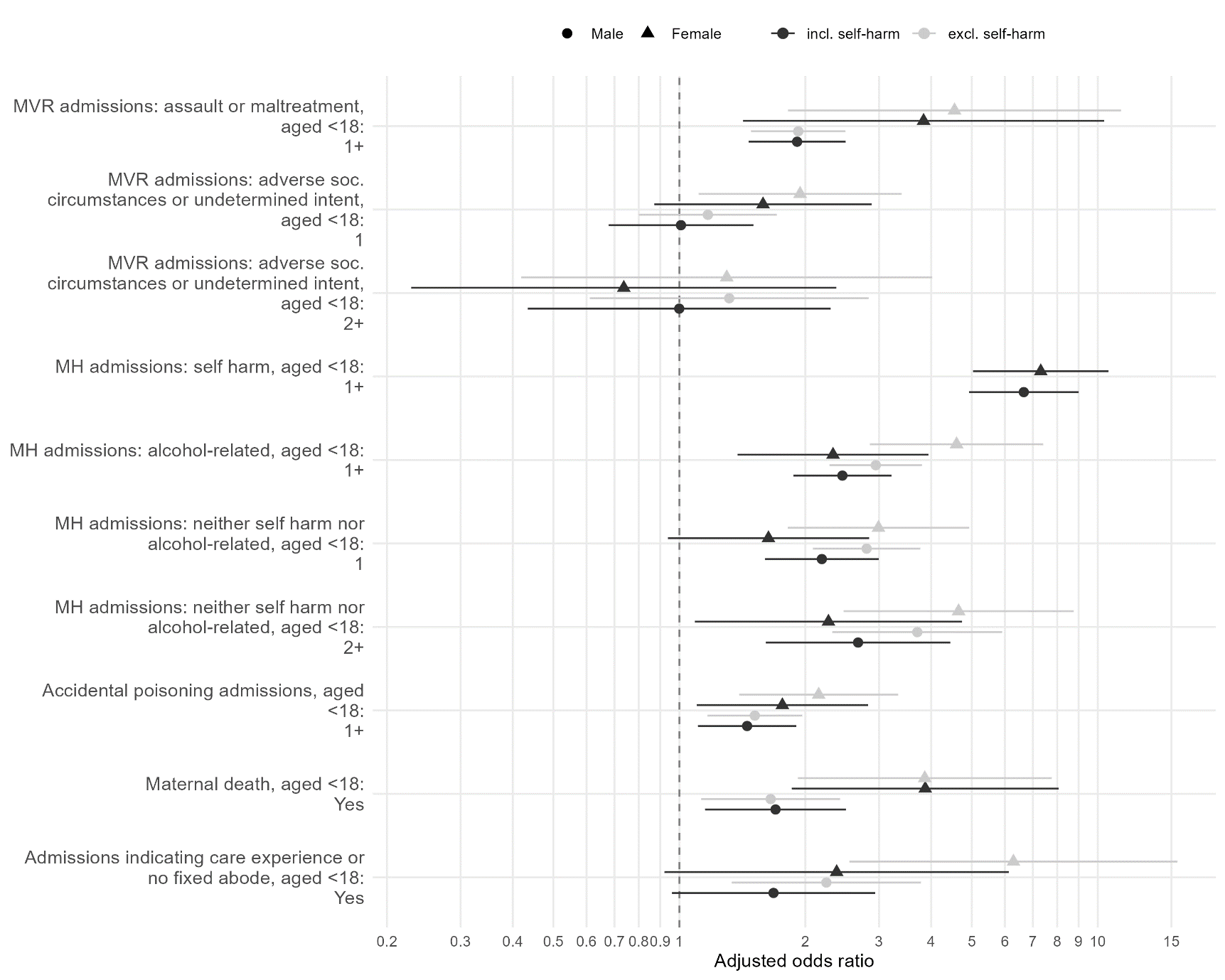


MVR: Maltreatment and violence-related; MH: mental health-related; Adjusted odds ratio (aOR) plot showing estimated effects of explanatory variable on case status (suicide) using conditional logistic regression (modified Model 1 with ‘subcategorised’ MVR and MH admissions), with estimates for male and female strata shown separately, as well as for models including or excluding self-harm admissions. Reference levels were having no respective admissions, or not having experienced maternal death. Horizontal line segments represent 95% confidence interval.

## Note on codes suggestive of maltreatment or neglect

Codes suggestive of maltreatment or neglect defined for ages up to 10 were relatively common for both cases, 307/2,106 (14.6%) and controls, 2,042/13,589 (15.0%), OR=0.96 (95% CI, 0.85-1.10), mostly due to the large proportion of dental caries-coded inpatient admissions that matched criteria suggestive of maltreatment or neglect; when dental caries was excluded from the criteria, the rate decreased to 74/2,106 (3.5%) for cases and 366/13,589 (2.7%) of controls, OR=1.32 (95% CI, 1.02-1.70). The OR was significant for females, OR=1.99 (95% CI, 1.20-3.28) but not for males, OR=1.17 (95% CI, 0.87-1.57); even after excluding dental caries, these effects were no longer significant once adjusted for the other adversities in the conditional logistic regression (Model 1): there was no association with suicide in either stratum with aOR_male_=0.92 (95%CI, 0.64-1.32) and aOR_female_=1.06 (95%CI, 0.50-2.23).
Unlike the 2011 Schnitzer et al. paper^6^ we omitted the use of dental caries codes as suggestive of neglect. In the original study, dental caries codes were included due to the “extensive number and advanced state of caries”, necessitating general anaesthesia. Their data combined emergency department (ED) and hospital admissions, with 79% of the cases reviewed rated to be possibly, rather than probably, suggestive of maltreatment or neglect – in the context of the other codes suggestive of maltreatment or neglect reviewed in the study, dental caries was in the lower end of the range of certainty. In the present study, approximately 80% of individuals matching criteria suggestive of neglect had dental caries-related diagnoses or procedures, and only a small proportion included procedure codes for anaesthesia and/or multiple sites of caries. In Scotland, dental procedures in SMR01 (acute inpatient admissions) partly represent Community Dental Services, which provide general dentistry in areas with few General Dental Practitioners (e.g. rural areas) and to particular populations e.g. patients with physical disabilities, learning disabilities, or long-stay care residents^7^. In the absence of case note review it is unlikely that the frequent dental caries cases observed in Scottish inpatient records indicate severe enough cases to be suggestive of neglect.

# Details of conditional logistic regression

Two multiple conditional logistic regression models were computed, stratified by sex. Model 1 included MVR admissions, MH admissions, admissions suggestive of maltreatment or neglect excluding dental caries, accidental poisoning admissions, maternal death, and admissions indicating care experience or no fixed abode. For reasons of Statistical Disclosure Control, the number of admissions for each type of adversity or MH category were categorised such that no category included fewer than 10 individuals. MH admissions were categorised into having 0, 1, 2, or 3 or more admissions; MVR admissions were categorised in to having 0, 1 or 2 or more admissions; accidental poisonings were categorised into having zero or one admission as were admissions indicating care experience or no fixed abode.

Model 2 was intended to investigate the interaction between adverse events and MH admissions. A single count variable of the types of adversity experiences was defined, with types defined as: MVR admissions, accidental poisoning, maternal bereavement, and codes indicating care experience or no fixed abode; the count of the number of adversity types was used alongside the number of MH admissions, and their interaction term, as explanatory variables.

An additional model based on Model 1 was also computed, with MVR admissions further divided into admissions containing codes for “Assault or Maltreatment”, and “Adverse social circumstances or Events of undetermined intent”; MH admissions were divided into self-harm admissions, alcohol-related admissions, and admissions that were neither alcohol- nor self-harm-related (NANSHR), to investigate which of those types of adversity had the largest effect. To ensure there were at least 10 individuals in each category, NANSHR MH admissions and admissions with Adverse social circumstances or Events of undetermined intent were the only categories permitting categorising individuals into having 0, 1, or 2+ admissions; for all other categories, multiple admissions were sparse and so we distinguished only between zero and one or more admissions.

All adverse admissions were defined as occurring before age 18, with the exception of admissions suggestive of maltreatment or neglect, which were defined for ages under 10. Sex, age, and geographical variables were used for case-control matching and were not included in the regression models. After removing individuals with no hospitalisations prior to death (for controls, no hospitalisations before the date equivalent to the death of their matched case) there were N=3,559 individuals in the female stratum and N=12,136 individuals in the male stratum. To balance the sparsity of the data with not producing disclosive numbers, the number of admissions in each adversity type was discretised so that the smallest cell count in each stratum was at least 10 after accounting for missing values. No multicollinearity was detected using a generalised Variance Inflation Factor (GVIF) computation^8^, with a maximum ${GVIF}^{2\frac{1}{2Df}}$ as follows:

Supplementary Table 17: Generalised Variance Inflation Factor (GVIF)

| *Stratum* | Male | Female |
| --- | --- | --- |
| Model 1 | 1.39 | 1.73 |
| Model 1 with ‘subcategorised’ MVR & MH admissions | 1.41 | 1.68 |

## Controlling for geographical variables

After we excluded individuals with no hospital admissions or a single death-related admission, there was no longer a balance between matched cases and controls on geography, and there was in fact an association between the urban-rural indicator and case status, specifically an association between Large urban areas and cases. There was also an association between Urban-rural indicator and having hospital admissions prior to death, with those with no hospital admissions more concentrated in Large urban areas, especially for controls. Despite this, most cases and controls shared identical geographical variables, making them non-estimable in conditional logistic regression.

## A note on (non-)overlapping adversity categories in logistic regression model

Our aim was to create a wide set of inclusion criteria indicating childhood adversity to maximise sensitivity. Because of this, there was some overlap between codes included in the adversity types: Maltreatment or violence-related (MVR) codes^9,10^, mental health-related codes (using CCS coding^11,12^), and Codes suggestive of maltreatment^6^. In particular, *Undetermined intent* codes were included in both MVR, CCS, and Codes suggestive of maltreatment criteria, though in practice the overlap was small due to the low number of admissions containing those codes. Similarly, intentional poisoning was included as part of codes denoting Self-injury in the CCS codes (Mental health-related codes), so only accidental poisoning was included as an explanatory variable in the regression model. For computing univariate statistics, the same diagnosis code could count towards multiple adversity types (e.g. a person with an appropriate code at admission could be counted against MVR diagnoses, CCS diagnoses, and codes suggestive of maltreatment). For the regression model, the above listed adversities were made mutually exclusive at the level of diagnosis code in the same admission. Specifically, if the same diagnosis code in the same admission was already included under one set of adversity criteria, it was excluded from the others. The order for these was as follows: MVR admissions were computed first, followed by CCS (mental health-related) admissions, followed by admissions suggestive of maltreatment or neglect; an injury of undetermined intent would count as an MVR event in this case, despite matching the criteria for CCS and codes *suggestive* of maltreatment. Conversely, different diagnosis codes in the same admission were eligible for inclusion under multiple adversity criteria: an admission where both an assault code and a mental health code were present would have counted both towards MVR, and mental-health related code (CCS) criteria, for example.

## Note on variable selection

The final model presented was the ‘full’ model including all hypothesised adversities and mental health admissions, with the aim of demonstrating the relative ranking of associations of the explanatory variables with suicide. Goodness-of-fit tests showed no significant improvement when non-significant explanatory variables were removed one at a time, so all variables were retained in the final model.

##

# References

1 National Records of Scotland. Probable Suicides: Deaths which are the Result of Intentional Self-harm or Events of Undetermined Intent. National Records of Scotland, 2020 /statistics-and-data/statistics/statistics-by-theme/vital-events/deaths/suicides (accessed Feb 25, 2021).

2 Office for National Statistics. Suicides in the UK Quality and Methodology Information. Office for National Statistics. 2019. https://www.ons.gov.uk/peoplepopulationandcommunity/birthsdeathsandmarriages/deaths/methodologies/suicideratesintheukqmi#other-information (accessed Aug 31, 2022).

3 WHO. International Statistical Classification of Diseases and Related Health Problems 10th Revision, Version 2019. 2019.

4 Pavis S, Morris AD. Unleashing the power of administrative health data: the Scottish model. *Public Health Res Pr* 2015; **25**. DOI:10.17061/phrp2541541.

5 Public Health Scotland, GPD Team. Deprivation guidance for analysts, version 3.4. 2020. https://www.isdscotland.org/Products-and-Services/GPD-Support/Deprivation/_docs/PHS-Deprivation-Guidance-version-3-4.pdf (accessed Aug 3, 2020).

6 Schnitzer PG, Slusher PL, Kruse RL, Tarleton MM. Identification of ICD codes suggestive of child maltreatment. *Child Abuse & Neglect* 2011; **35**: 3–17.

7 Dental Care | Community Dental Service | Health Topics | ISD Scotland. https://www.isdscotland.org/Health-Topics/Dental-Care/Community-Dental-Service/ (accessed Dec 2, 2021).

8 Fox J, Monette G. Generalized Collinearity Diagnostics. *Journal of the American Statistical Association* 1992; **87**: 178–83.

9 González-Izquierdo A, Woodman J, Copley L, *et al.* Variation in recording of child maltreatment in administrative records of hospital admissions for injury in England, 1997–2009. *Archives of Disease in Childhood* 2010; **95**: 918–25.

10 Gilbert R, Fluke J, O’Donnell M, *et al.* Child maltreatment: variation in trends and policies in six developed countries. *The Lancet* 2012; **379**: 758–72.

11 Elixhauser A, Steiner C, Palmer L. Clinical classifications software (CCS). Clinical Classifications Software (CCS). 2012. http:// www.hcup-us.ahrq.gov/toolssoftware/ccs/ccs.jsp.

12 US Agency for Healthcare Research and Quality. Clinical Classifications Software (CCS) for ICD-10. Clinical Classifications Software (CCS). 2019. https://www.hcup-us.ahrq.gov/toolssoftware/ccsr/ccs_refined.jsp.
